# Facet‐Controlled Synthetic Strategy of Cu_2_O‐Based Crystals for Catalysis and Sensing

**DOI:** 10.1002/advs.201500140

**Published:** 2015-08-14

**Authors:** Yang Shang, Lin Guo

**Affiliations:** ^1^ Key Laboratory of Bio‐Inspired Smart Interfacial Science and Technology, Ministry of EducationSchool of Chemistry and EnvironmentBeihang UniversityBeijing100191P.R. China; ^2^ Key Laboratory of Micro‐Nano Measurement‐Manipulation and Physics, Ministry of EducationSchool of Physics and Nuclear Energy EngineeringBeihang UniversityBeijing100191P.R. China

**Keywords:** catalysis, Cu_2_O, facet‐controlled, nanocrystals, sensing

## Abstract

Shape‐dependent catalysis and sensing behaviours are primarily focused on nanocrystals enclosed by low‐index facets, especially the three basic facets ({100}, {111}, and {110}). Several novel strategies have recently exploded by tailoring the original nanocrystals to greatly improve the catalysis and sensing performances. In this Review, we firstly introduce the synthesis of a variety of Cu_2_O nanocrystals, including the three basic Cu_2_O nanocrystals (cubes, octahedra and rhombic dodecahedra, enclosed by the {100}, {111}, and {110} facets, respectively), and Cu_2_O nanocrystals enclosed by high‐index planes. We then discuss in detail the three main facet‐controlled synthetic strategies (deposition, etching and templating) to fabricate Cu_2_O‐based nanocrystals with heterogeneous, etched, or hollow structures, including a number of important concepts involved in those facet‐controlled routes, such as the selective adsorption of capping agents for protecting special facets, and the impacts of surface energy and active sites on reaction activity trends. Finally, we highlight the facet‐dependent properties of the Cu_2_O and Cu_2_O‐based nanocrystals for applications in photocatalysis, gas catalysis, organocatalysis and sensing, as well as the relationship between their structures and properties. We also summarize and comment upon future facet‐related directions.

## 1 Introduction

In addition to the shapes of nanocrystals (NCs), their surface conditions (surface energies and electronic structures) also determine their physical and chemical properties.^1^ Facets with distinctive crystallographic feature possess different atomic terminated characters, which have shown big differences in catalysis and sensing.^2^, ^3^, ^4^, ^5^, ^6^, ^7^, ^8^, ^9^ Over the past decades, the understanding, design, and optimization of metal oxide NCs enclosed by well‐defined facets has been widely explored.^7^, ^10^, ^11^, ^12^, ^13^, ^14^, ^15^, ^16^ It is noteworthy that, although high‐index facets that have high‐density atomic edges with corners and plentiful unsaturated active sites are promising for catalysis and sensing applications, those facets are often unstable, and hardly obtained by traditional chemical methods.^17^, ^18^, ^19^, ^20^, ^21^, ^22^, ^23^ Thus, the shape‐dependent catalysis and sensing behaviours is primarily focused on NCs enclosed by low‐index facets, especially the three basic facets (i. e., the {100}, {111}, and {110} facets).^6^, ^7^, ^8^, ^9^, ^12^, ^13^, ^14^, ^16^, ^24^, ^25^, ^26^, ^27^, ^28^, ^29^, ^30^, ^31^, ^32^, ^33^, ^34^ For instance, by employing hydrofluoric acid as a capping agent (CA), H. G. Yang et al.^34^ were the first to obtain uniform anatase TiO_2_ single crystals with a high percentage (47%) of highly reactive {001} facets, which possessed promising applications in sensors, solar cells and photocatalysis. Besides the various routes for the synthesis of NCs, several novel strategies have recently exploded by carving, modifying, or transforming the original NCs that greatly improve the catalysis and sensing performances.^6^, ^27^, ^30^, ^33^, ^35^, ^36^, ^37^, ^38^, ^39^, ^40^, ^41^, ^42^, ^43^, ^44^, ^45^, ^46^, ^47^, ^48^, ^49^, ^50^, ^51^, ^52^ For example, X. Chen et al.^51^ disordered the surface layers of nanophase TiO_2_ by hydrogenation. The disorder‐engineering substantially improved the solar photocatalytic performances of TiO_2_. R. Long et al.^30^ fabricated a metal–semiconductor hybrid structure in which Pd nanocubes exposed with {100} facets were deposited on TiO_2_ supports. By changing the light intensity irradiated on Pd–TiO_2_ heterogeneous structures, the charge condition of the Pd surface could be rationally modulated, and thus the function of Pd nanocubes in organic oxidation reactions and O_2_ activation could be tailored. Our group^35^ synthesized Ni–Co amorphous double hydroxides nanocages with tunable Ni/Co molar ratio by using Cu_2_O octahedra as templates. The obtained amorphous NiCo_2.7_(OH)*_x_* nanocages displayed outstanding applications in electrochemical water oxidation.

In this context, the inexpensive, non‐toxic and abundantly available Cu_2_O nanomaterials, with unique optical and electrical properties,^4^, ^9^, ^10^, ^53^, ^54^, ^55^, ^56^, ^57^, ^58^ have recently aroused general attention, due to their outstanding morphology‐dependent applications in catalysis (gas oxidation,^2^, ^3^, ^17^, ^59^, ^60^, ^61^ CO_2_ reduction,^62^, ^63^, ^64^, ^65^ organocatalysis,^14^, ^24^, ^32^, ^40^, ^50^, ^66^ electrocatalysis,^28^, ^67^, ^68^ and photocatalysis^36^, ^44^, ^69^, ^70^, ^71^, ^72^, ^73^, ^74^, ^75^, ^76^, ^77^, ^78^, sensing (gas sensors,^8^, ^79^, ^80^, ^81^, ^82^, ^83^ ion detection,^29^ and surface‐enhanced Raman scattering (SERS)^84^, ^85^, ^86^, ^87^, ^88^, ^89^, as adsorbents,^7^, ^90^ biotoxicity,^26^, ^91^, ^92^ as chemical templates^35^, ^38^, ^45^, ^47^, ^48^, ^49^, ^52^, ^93^, ^94^, ^95^, ^96^ and energy‐related processes (water splitting,^97^, ^98^, ^99^, ^100^ solar energy conversion^101^, ^102^ and lithium‐ion batteries^25^, ^103^. Compared to Cu_2_O nanowires^72^, ^81^, ^104^, ^105^, ^106^ or nanorods,^83^, ^107^ nanospheres,^53^, ^64^, ^76^, ^82^, ^84^, ^87^, ^90^, ^108^, ^109^ hollow structures,^42^, ^57^, ^67^, ^73^, ^79^, ^94^, ^95^, ^101^, ^110^, ^111^, ^112^, ^113^, ^114^ self‐assembled superstructures,^8^, ^115^, ^116^ and Cu_2_O polyhedra enclosed by high‐index planes,^17^, ^117^, ^118^, ^119^, ^120^, ^121^, ^122^ the preparation of Cu_2_O polyhedra enclosed by low‐index planes^2^, ^3^, ^7^, ^8^, ^9^, ^14^, ^25^, ^26^, ^29^, ^32^, ^55^, ^57^, ^66^, ^68^, ^75^, ^91^, ^92^, ^123^, ^124^, ^125^, ^126^, ^127^, ^128^, ^129^ is simple and large‐scale. Even more importantly, several novel facet‐controlled routes, including carving,^42^, ^44^, ^110^, ^130^, ^131^, ^132^ modifying^36^, ^40^, ^50^, ^70^, ^73^, ^133^ and converting,^35^, ^38^, ^45^, ^47^, ^48^, ^49^, ^52^, ^60^, ^93^, ^134^ have been recently carried out on the basis of the well‐defined facets of Cu_2_O NCs, especially in cubic, octahedral, and rhombic dodecahedral crystals (the three basic Cu_2_O crystals, enclosed by the {100}, {111}, and {110} low‐index facets, denoted as *c*‐Cu_2_O, *o*‐Cu_2_O and *d*‐Cu_2_O, respectively), to tailor their facet‐dependent properties. It is noteworthy that although smaller NCs possess higher activities in catalysis and sensing than larger NCs, the reported activity of of smaller Cu_2_O NCs^8^, ^32^, ^68^ remain ≈1 order of magnitude lower than those of larger Cu_2_O NCs; thus, larger Cu_2_O NCs are often used as precursors for further carving, modifying and transforming.

In this review, we comprehensively summarized the recent progresses in facet‐controlled synthetic strategies for the preparation of Cu_2_O‐based NCs as well as tailoring their facet‐dependent properties of catalysis and sensing. We begin with a brief discussion of solution phase synthetic strategy of the three basic Cu_2_O NCs (*c*‐Cu_2_O, *o*‐Cu_2_O and *d*‐Cu_2_O) and Cu_2_O NCs enclosed by high‐index planes, as well as the key role of CA for controlling their crystallographic facets. We then introduce in detail the three main facet‐controlled synthetic strategies (deposition, etching and template) on the Cu_2_O NCs to fabricate Cu_2_O‐based NCs with heterogeneous, etched, or hollow structures, and discuss in detail a number of important concepts involved in those facet‐controlled routes, including the selective adsorption of CA for protecting special facets, and the impacts of surface energy and active sites on reaction activity trends. Finally, we summarize the exciting facet‐dependent properties of Cu_2_O and Cu_2_O‐based NCs for applications of photocatalysis, gas catalysis, organocatalysis and sensing, as well as the relationship between their structures and properties. We expect that this review will inspire facet‐controlled methodologies, and more examples of these facet‐dependent properties should be continuously explored, endowing nanomaterials with excellent properties for numerous applications.

## 2 Basic Growth Strategies for Cu_2_O Polyhedra

**Figure 1:**
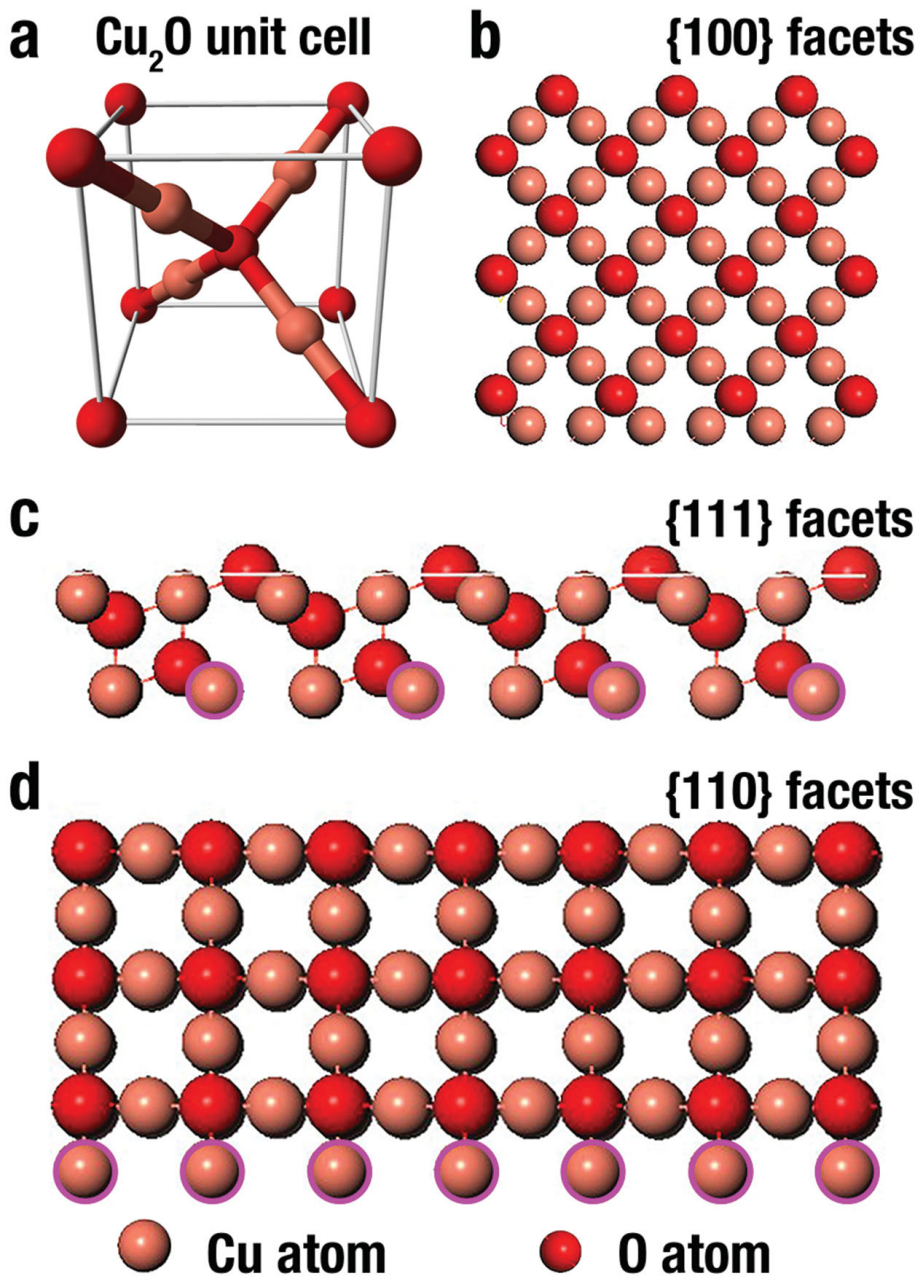
Illustration of the a) unit cell of cuprite Cu_2_O, and b–d) the crystal structure of Cu_2_O {100}, {111} and {110} facet, respectively. The light pink spheres are Cu atoms and the red spheres are O atoms. The dangling Cu atoms are marked by dark pink circles.

Cu_2_O crystallizes in a cubic structure. A tetrahedron of Cu atoms encircle every O atom, and every Cu atom possesses two neighboring O atoms as illustrated in the model of unit cell^123^ (**Figure** **1**a). For the {100}, {111} and {110} facets, the three low‐index facets of Cu_2_O crystals, it is well established that the surface energy is closely related to the density of under‐coordinated Cu atoms.^75^ The atomic arrangements along three low‐index facets of Cu_2_O are illustrated in Figure 1b– 1d. Only O atoms are terminated in the {100} facet, leading to electric neutrality (Figure 1b).^44^ By contrast, Cu atoms at the {111} facet are coordinated unsaturated. Each two Cu atoms have a dangling bond perpendicular to the {111} facet illustrated by the pink circles in Figure 1c, which make them positively charged.^7^ Similarly, the {110} facet has the same terminated Cu atoms with dangling Cu atoms (illustrated by the pink circles in Figure 1d), while the number of dangling Cu atoms on {110} plane per unit surface area is approximately 1.5 times higher than that on {111} plane.^75^ Thus, the {110} facet should be more positively charged than the {111} facet, and the surface energies of Cu_2_O are in the following order: *γ*_{100}_ < *γ*_{111}_ < *γ*_{110}_.

**Figure 2:**
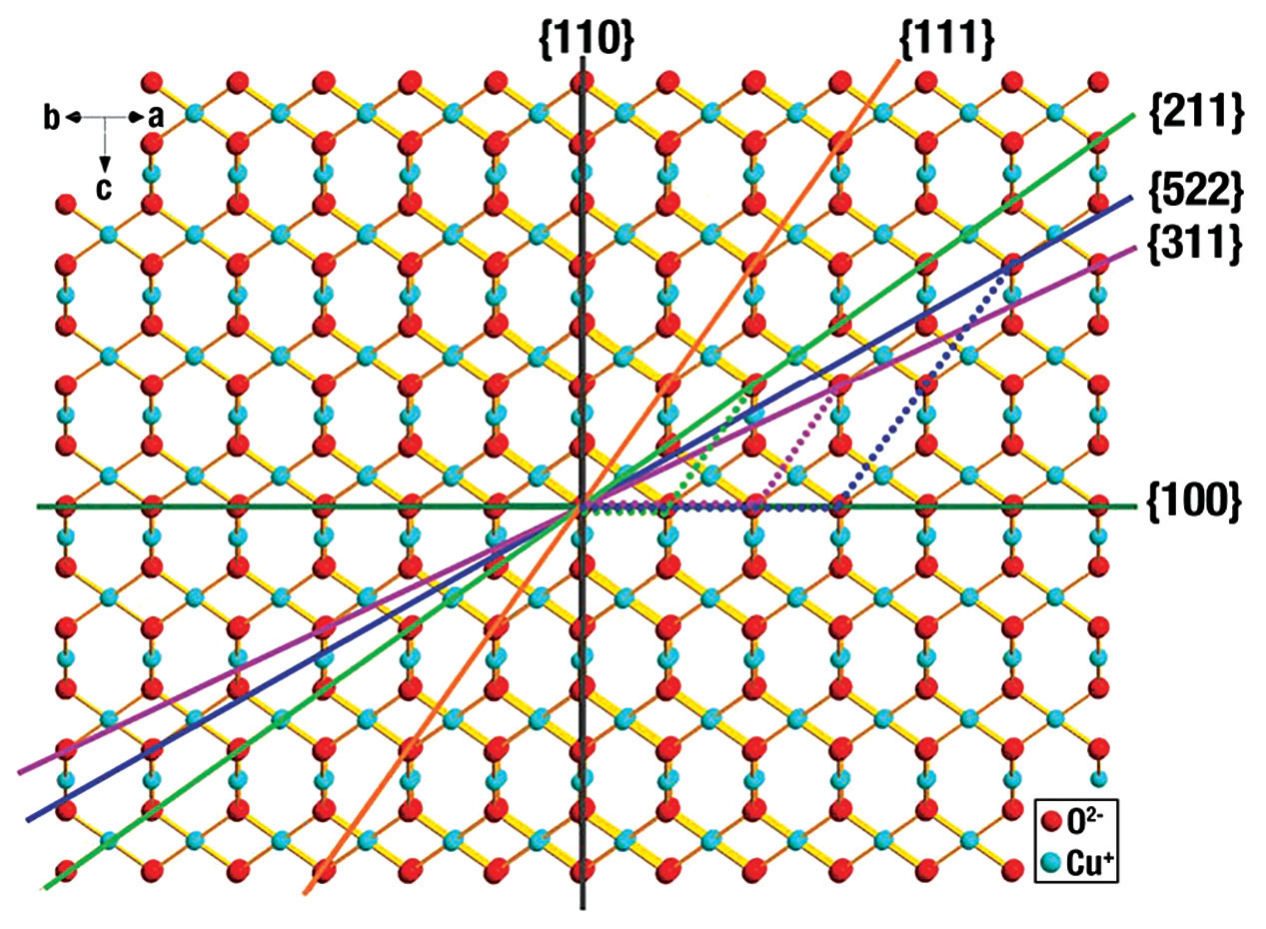
Illustration of the crystal structure of Cu_2_O viewed from the direction parallel to the {110} facets. Reproduced with permission.^135^ Copyright 2012, Royal Society of Chemistry.

However, the conditions of high‐index facets of Cu_2_O NCs are distinctly different. For example, the {311}, {522} and {211} facets can be displayed by a terrace × step as 2 {100} × {111}, 3 {100} × 2 {111}, and {100} × {111}, respectively. That is, they possess two {100} terraces and one {111} step, three {100} terraces and two {111} steps, and one {100} terrace and one {111} step, respectively (**Figure** **2**).^135^ Therefore, compared to low index {100} and {111} facets, the numerous kinks and steps endow those high‐index facets with higher surface energies.

**Figure 3:**
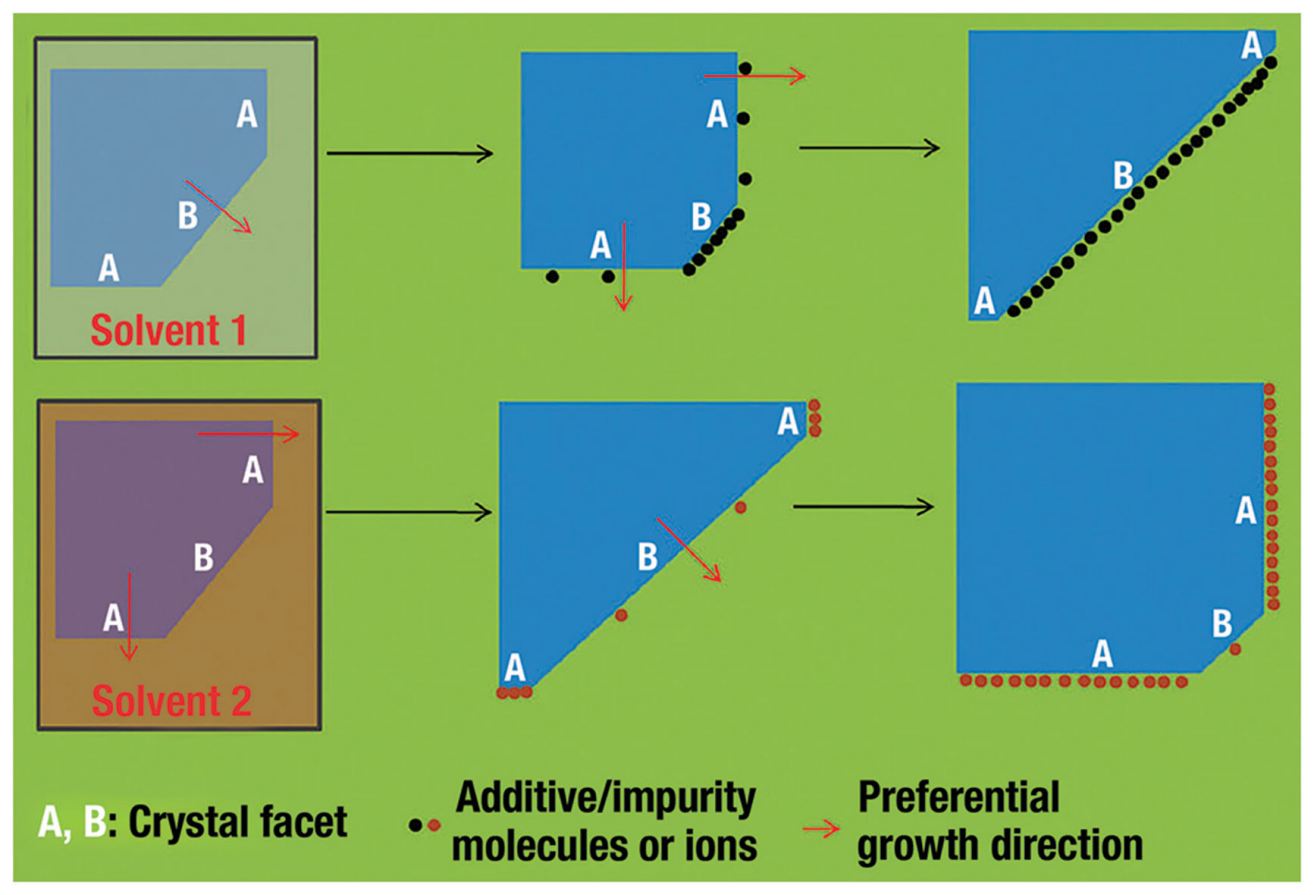
Illustration of facet‐control of crystal facets by solvent and additive/impurity molecules or ions. Reproduced with permission.^5^ Copyright 2011, Royal Society of Chemistry.

Cu_2_O crystals with clean facets were primarily synthesized through solution phase synthesis (hydrothermal and solvothermal process),^4^, ^7^, ^8^, ^17^, ^44^, ^75^, ^101^, ^107^, ^122^, ^124^ because that route could delicately tailor the exposed facets of crystals, through controlling the nucleation and growth behaviours (especially growth rates in different directions) of crystals.^136^, ^137^ The Wulff construction determines the equilibrium or natural morphologies of crystals, because minimizing the total surface energies mainly lead the shape evolution of crystal.^123^ Based on the Gibbs–Wulff's theorem, the facets with higher surface energies always grow rapidly and finally decrease or vanish from the ultimate morphologies, while the crystal facets with lower surface energies grow slowly and are preserved in the final structure.^5^ However, selective surface stabilization of appropriate organic or inorganic additives (molecules or ions) as CA can effectively decrease the surface energies and retard the crystal growth along their normal orientations (**Figure** **3**).^5^, ^7^ CAs tend to selectively adsorb on the surface with higher surface energy, which consequently lead to delicately tuning of the percentages of different facets of crystals.^7^, ^138^

**Figure 4:**
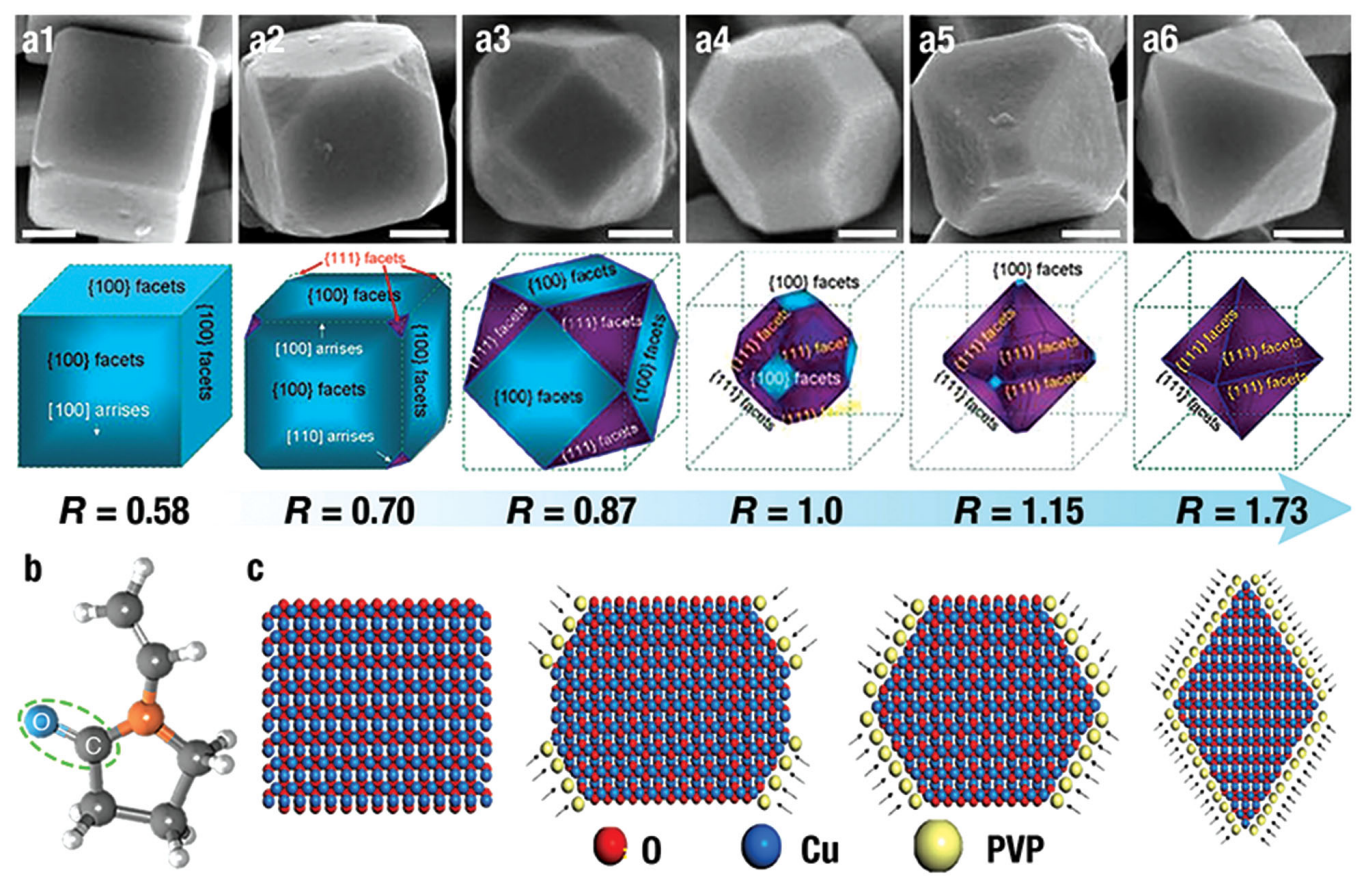
a) SEM of the Cu_2_O polyhedral NCs depending of the ratio *R* (the ratio of the growth rate along <100> to that of <111>), and the corresponding 3D structures. Scale bar = 300 nm. b) The molecular formula of PVP. c) PVP adsorption during the growth process of Cu_2_O NCs. Reproduced with permission.^7^ Copyright 2009, Royal Society of Chemistry.

**Figure 5:**
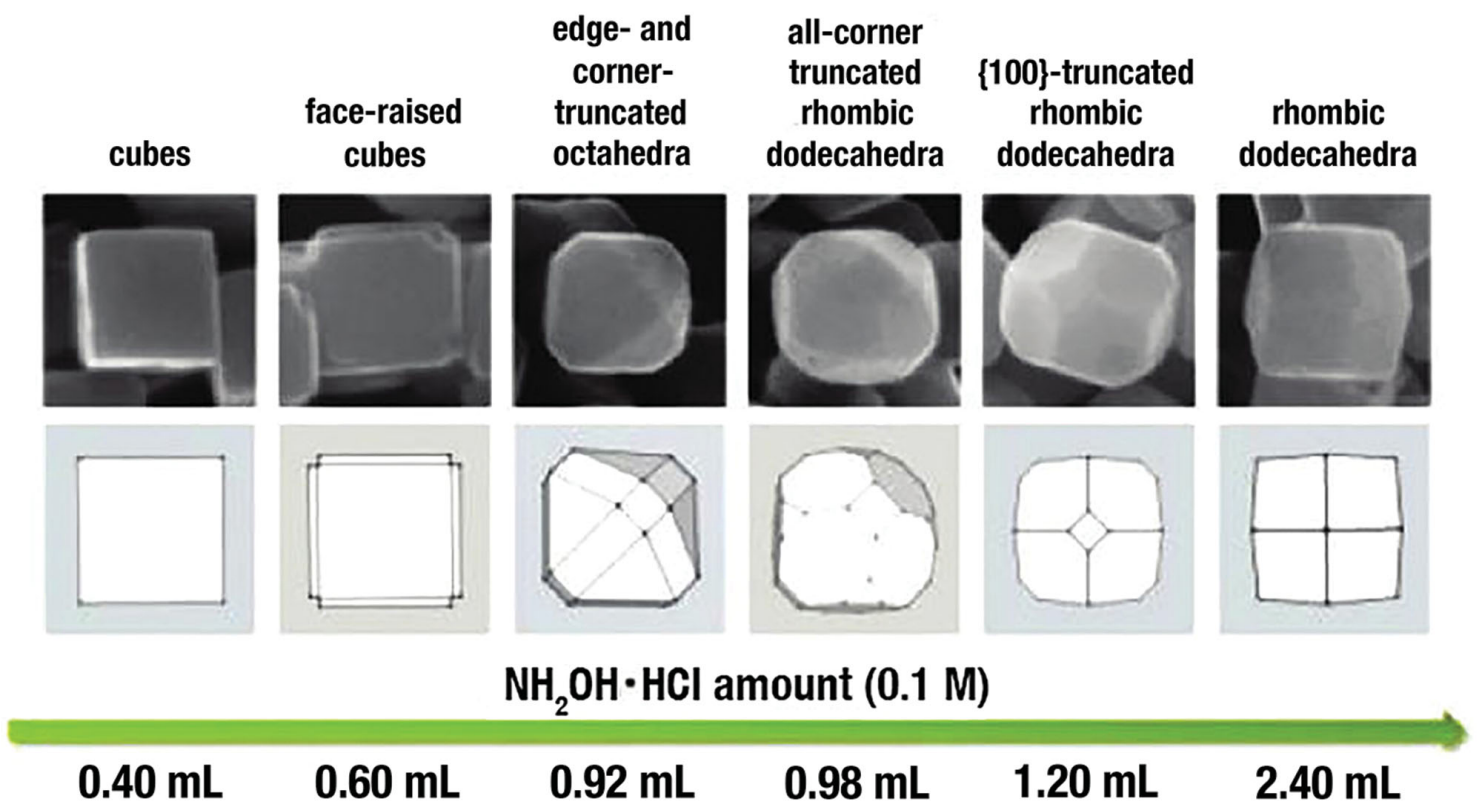
SEM images and the corresponding geometry models with shape evolution from *c*‐Cu_2_O to *d*‐Cu_2_O. Reproduced with permission.^75^ Copyright 2012, American Chemical Society.

To date, CAs have played an important role on shape‐controlled synthesis of NCs,^137^, ^138^, ^139^, ^140^, ^141^, ^142^ and there are many successful examples in preparing Cu_2_O NCs.^7^, ^8^, ^75^, ^107^, ^122^, ^125^, ^143^ We will introduce some classic synthetic routes of Cu_2_O NCs enclosed by low‐index facets. For instance, by using the preferential adsorption of polyvinylpyrrolidone (PVP) on the {111} facets, our group^7^ successfully achieved the systematic morphology evolution from *c*‐Cu_2_O to *o*‐Cu_2_O (**Figure** **4**a), which was in accordance with the identical evolution in shapes of cubic‐structured crystal depending on the ratio *R* (the growth rate ratio of <100> to <111>).^144^ The negatively charged O atoms of “—C = O” in PVP (Figure 4b) would strongly interact with the positively charged dangling Cu atoms on {111} facet to stabilize the crystal surfaces. The ratio of the surface area of {111} to {100} could be controlled by increasing the concentration of PVP (Figure 4c). It is worth noting that {110} facets could not be obtained by only using PVP as CA. The reason is that the relatively strong adsorption of PVP is not enough to reduce the growth rate of {110} facets. Interestingly, L. Gao et al.^125^ reported that, by employing oleic acid with stronger adsorption ability as the CA, rhombic dodecahedron Cu_2_O NCs totally enclosed by {110} facets could be obtained. With the increasing concentrations of oleic acid, the morphologies of Cu_2_O crystals were evolved from *c*‐Cu_2_O, *o*‐Cu_2_O, {110} truncated *o*‐Cu_2_O, to *d*‐Cu_2_O. During this process, oleic acid firstly adsorbed on the {111} facets to form *o*‐Cu_2_O; with continuous increasing of the concentration of oleic acid, the oleic acid began to adsorb on the {110} crystal planes. The area of the {110} surface was ever increasing, while the {111} surfaces gradually disappeared. Finally, *d*‐Cu_2_O enclosed by {110} facets were synthesized. By using sodium dodecyl sulfate (SDS) as CA, M. H. Huang et al.^75^ synthesized a succession of Cu_2_O NCs with morphology evolution from *c*‐Cu_2_O to *d*‐Cu_2_O (**Figure** **5**). The adding NH_2_OH·HCl played a dual role in reducing Cu(OH)_2_ to Cu_2_O and controlling the pH. By increasing the amount of NH_2_OH·HCl, the gradually decreased solution pH that caused by the HCl released from NH_2_OH·HCl would retard the formation rate of Cu_2_O. The rate for growing *c*‐Cu_2_O was within 1 min, but that for *d*‐Cu_2_O was decreased to 60 min.

This suggested that a slower growth rate contributed to the generation of *d*‐Cu_2_O. Furthermore, a slower growth rate, namely a kinetic‐controlled process is essential for obtaining high‐index facets. C. Wang et al.^17^ reported 50‐facet Cu_2_O polyhedral microcrystals partially enclosed by {311} high‐index facets. A low concentration of copper salts as well as a weak reducing agent contributed to the kinetic‐controlled process, and the decreased viscosities caused by the extra ethanol may improve the diffusions of the reactants. Those above factors finally contributed to the generation of the novel configurations. So far, another shape of 50‐facet Cu_2_O architectures with {311}, {522}, {211} facets,^135^ 50‐facet and 74‐facet Cu_2_O polyhedra with {211}, {522} and {744} facets^117^ and 30‐facet Cu_2_O polyhedra with {332} facets^121^ could also be obtained through different kinetic‐controlled process by changing the concentration of reactants.

To sum up, by using CA or kinetic‐controlled process, Cu_2_O polyhedra with smooth surfaces could be easily obtained, which lays a solid foundation for further tailoring and investigation of the facet‐dependent performance.

## 3 Facet‐Controlled Deposition

Recently, numerous studies are focused on the formation of heterogeneous structures by rational growing supported substances (typically noble metal nanoparticles) on the support (typically metal oxides), since metal oxides can not only serve as a support for a better dispersibility of disperse metal nanoparticles (NPs), but also enhance the catalytic abilities by interacting with the metal NPs.^6^, ^27^, ^30^, ^33^, ^36^, ^40^, ^50^, ^61^, ^145^, ^146^, ^147^ Despite many successful examples on the synthesis of heterogeneous structures, it is noteworthy that the spatially controllable deposition of noble metal NPs on metal oxide support is a significant topic. For example, R. G. Li et al.^27^ demonstrated that for the monoclinic BiVO_4_ enclosed by {110} and {010} facets, photogenerated holes and electrons were transferred to the {110} and {010} surfaces for oxidation and reduction reactions respectively, due to the different energy levels of the two facets. When MnO*_x_* (oxidation co‐catalyst) and Pt (reduction co‐catalyst) were preferentially deposited by light‐induced deposition onto the {110} and {010} facets of BiVO_4_, the performances of photocatalytic water splitting were significantly improved. They further optimized the experiments to design two highly efficient photocatalyst systems (M/MnO*_x_*/BiVO_4_ and M/Co_3_O_4_/BiVO_4_, where M stands for noble metals).^6^ Besides the intrinsic nature of separation of charge between the two facets, the synergetic effect of those catalysts also played a significant role in enhancing photocatalytic performances. So far, lots of Cu_2_O‐based heterogeneous structures have been reported,^33^, ^36^, ^40^, ^50^, ^61^, ^70^, ^76^, ^77^, ^78^, ^89^, ^108^, ^148^ and the synthetic routes mainly focused on light‐induced deposition^33^, ^70^ or galvanic deposition.^28^, ^36^, ^40^, ^50^, ^76^, ^89^, ^148^ In this section, we plan to discuss the site‐selective deposition of noble NPs on the preferential faces, edges, or corners of Cu_2_O crystals.

**Figure 6:**
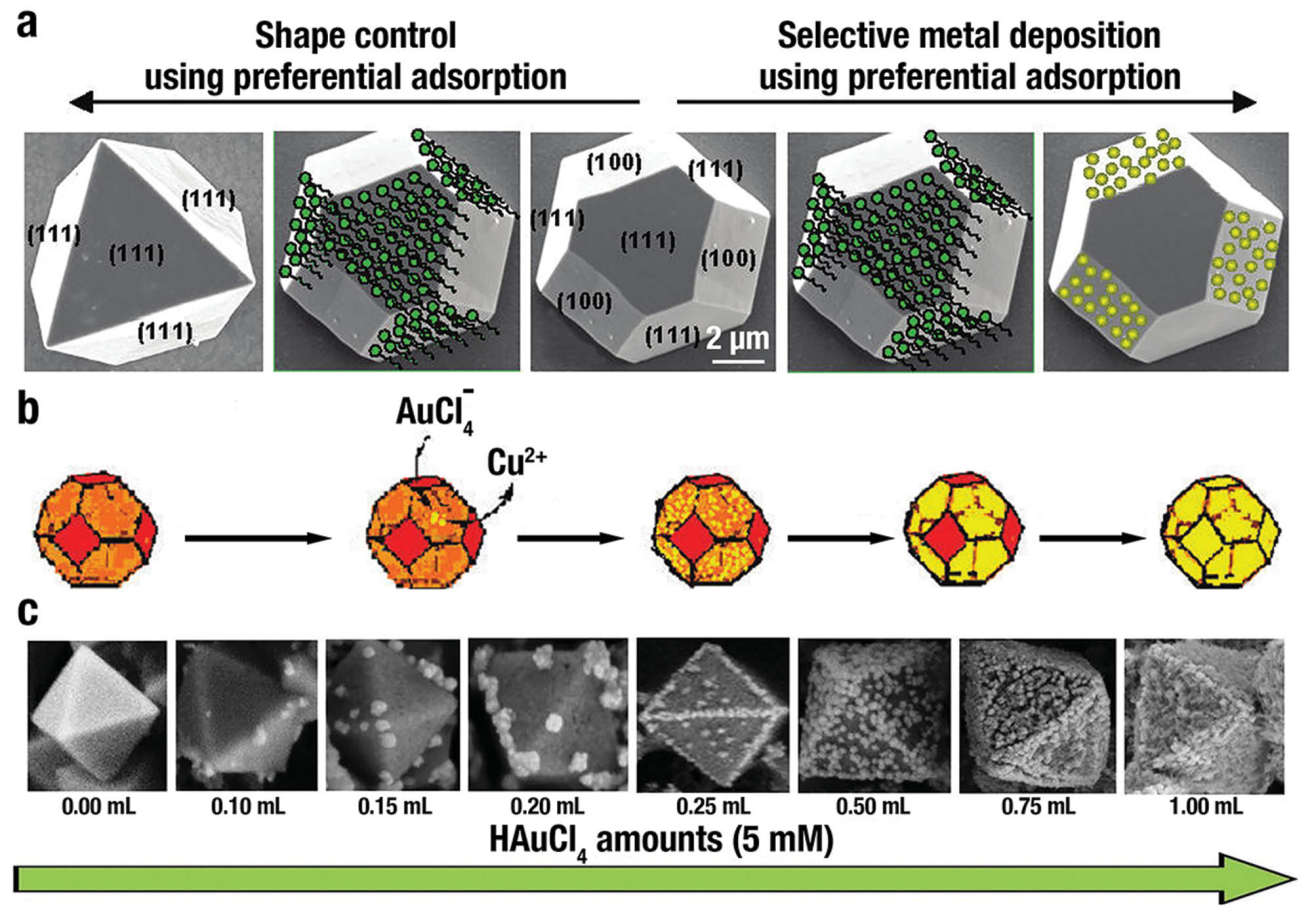
a) Using selective adsorption of SDS for controlling morphology (left) and for Au preferential deposition (right). Reproduced with permission.^133^ Copyright 2009, American Chemical Society. b) Selective growth of Au NPs on {111} facets of Cu_2_O microcrystals. Reproduced with permission.^28^ Copyright 2011, American Chemical Society. c) Illustration of the shape evolution of the preferential growth of Au NPs on *o*‐Cu_2_O. Reproduced with permission.^148^ Copyright 2013, Royal Society of Chemistry.

K. S. Choi et al.^126^ synthesized *o*‐Cu_2_O by employing the preferential adsorption of SDS on {111} facets (**Figure** **6**a, left). They then demonstrated that the selective adsorption of SDS could be used for preferentially blocking the nucleation of Au NPs on these planes (Figure 6a, right).^133^ In the presence of SDS, Au NPs only electrodeposited on the {100} facets of truncated octahedral Cu_2_O; however, Au NPs would form on both {100} and {111} facets in the absence of SDS.

By contrast, galvanic or light‐induced process can control the site‐selective deposition in the absence of CAs. X. W. Liu^28^ reported that during the in situ reduction of AuCl_4_^−^ precursors, a galvanic process occurred that Au NPs selectively grew on {111} facets of Cu_2_O truncated octahedra and cubooctahedra (Figure 6b), which can be formulated as shown in Eq. (1): (1)
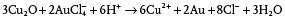



The galvanic deposition selectively occured on the {111} facet of Cu_2_O since that metallic component prefers to nucleate on highly active surface sites or defects with a large curvature, in which the {111} facet is more active than {100} facet due to the higher surface energy. By changing the concentration of the AuCl_4_^−^ precursor, the density and size of Au NPs can also be controlled. Unlike galvanic deposition, light‐induced deposition can lead to a distinct selectivity. After light illumination, the photogenerated electrons preferred to transfer from bulk to {100} surface of *c*‐Cu_2_O, which was contributed to reduce metal ions to pure metal. In contrast, the photoexcited holes mostly accumulated on the {111} facet that inhibited the reduction of metal ions.^33^

Edges and corners with a large curvature also play a key role in selective growth. M. L. Du et al.^148^ reported a sequential growth process of Au NPs on *o*‐Cu_2_O. With the increasing concentration of AuCl_4_^−^ ions, Au NPs were sequentially deposited on the corners, edges and facets of *o*‐Cu_2_O (Figure **6**c). The surface energy distribution follows the order of corners > crystal edges > {111} facets, and results in selective growth and evolution of the heterogeneous structures.

## 4 Facet‐Controlled Etching

Recently, much effort is dedicated to a so‐called “top‐down” engineering approach that delicately modifies crystals to create more highly active sites by etching and crystal cut, for the purpose of improvement the physical and functional properties of crystals.^43^, ^46^, ^51^, ^68^, ^149^, ^150^, ^151^, ^152^, ^153^, ^154^ (In this section, the “top‐down” means crystal carving without phase transformation; while the “top‐down” in the next section refers to total phase transformation from Cu_2_O to various hollow structures.) To date, various metal or alloys (Ag,^151^ Rh,^152^ Pd,^153^ Pd‐Pt,^139^ Pt,^155^ and Pt*_x_*Ni*_y_* 150 etc.) and metal oxide (Cu_2_O,^42^, ^44^, ^60^, ^66^, ^85^, ^110^, ^113^, ^130^, ^132^ TiO_2_,^13^, ^51^ Fe_2_O_3_,^46^ and ZnO^154^ etc.) NCs with sophisticated structures have been fabricated through a chemical “top‐down” route. The first step is partial dissolution of the mother‐crystal, namely via a surface etching process, in which the etching agent (ions or molecules) chelates to exposed facets by cations, and then leads the chelated surfaces to dissolve.^5^ A subsequent step of surface recrystallization on the residual surfaces of mother‐particles may occur, which make the mother‐particles roughen or convert to more stable facets.^5^ In other words, if the surface recrystallization process does not happen, the continuous surface etching process would contribute to the transformation from the mother‐crystal particles to hollow^42^, ^88^, ^110^, ^113^, ^114^, ^150^ or branch^41^, ^151^, ^152^, ^155^ structures.

In the absence of CAs, when many kinds of facets are exposed on the surface of a precursor, the etching will proceed with facet selectivity beginning with the facet(s) with the highest active sites. Although Ag_2_O has an identical cuprite crystal structure, the order of facet stability for Ag_2_O to chemical etching by NH_3_ and NaOH is {111} > {110} > {100}.^31^ The drastically different facet stability is caused by the pH of the reaction system. The {111} and {100} facet of Ag_2_O are terminated with Ag atoms and O atoms, respectively; while the {110} facet consists of rows of surface O and Ag atoms. Under alkaline condition, OH^−^ ions would strongly interact with Ag atoms on {111} facets and protect them from etching by NH_3_, while a protecting ionic layer does not exist for the {100} facets, leading to their dissolution by NH_3_. However, in an acidic environment, the {110} and {111} facets of Ag_2_O with high surface energy are unstable, and those facets would transform into {100} facets with low surface energy.

An appropriate CA (ions or molecules) could selectively adsorb on special facets to avoid dissolution. Thus, the existing CA is a critical factor when inferring the reacted facet in the initiation of an etching process. For example, using phosphate ions as CA selectively protected the {110} facet of Fe_2_O_3_ NCs; the etching by oxalic acid preferentially occurred along the [001] direction. Hence, the Fe_2_O_3_ NCs with minor {001} and major {110} facets would transform into Fe_2_O_3_ discs with minor {110} and major {001} facets.^46^ We intend to conclude the face‐dependent etching on Cu_2_O NCs, and study the formation mechanisms, including preferentialadsorption, etching, and others.

**Figure 7:**
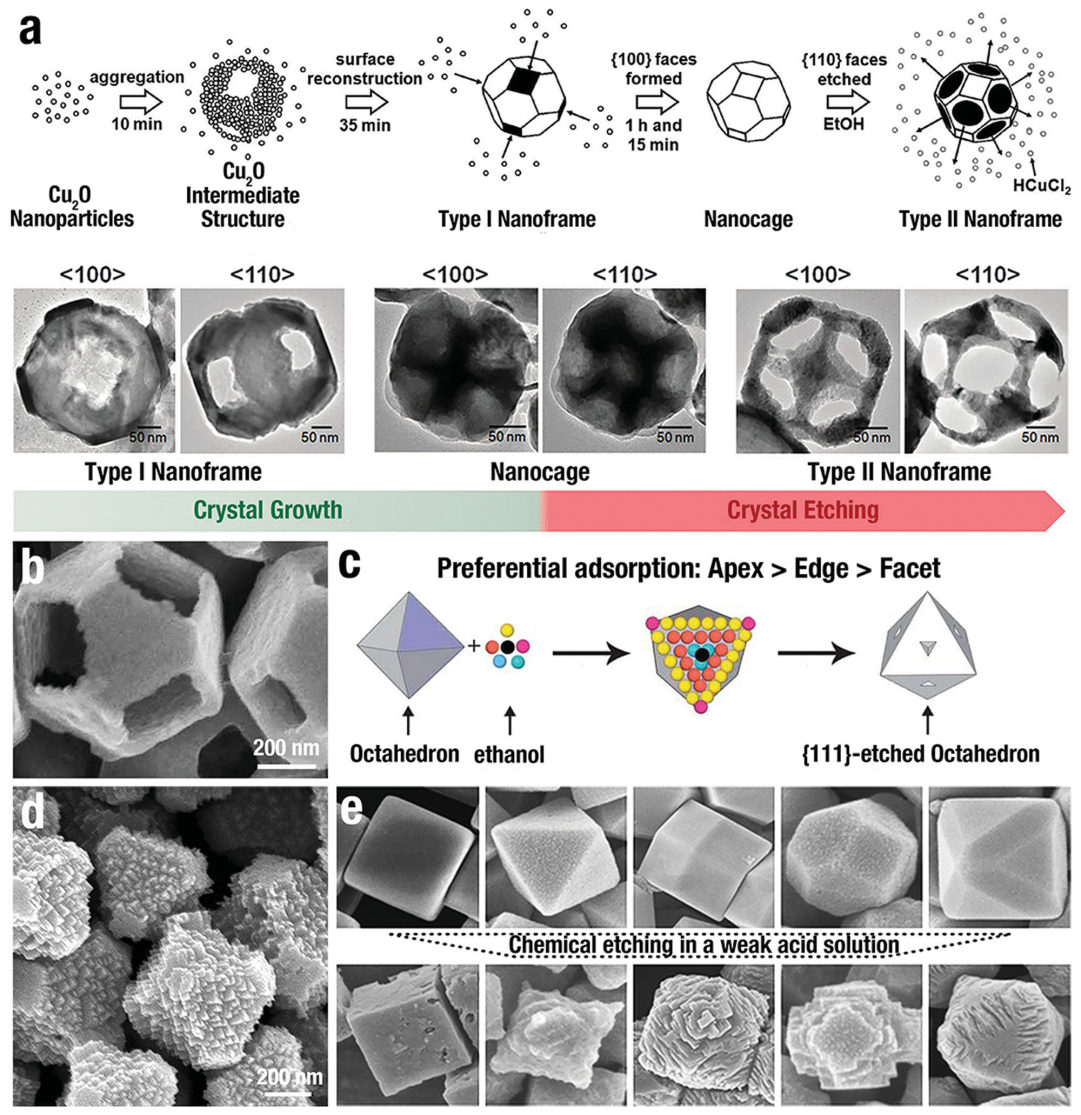
a) Growth schematic and SEM images of Cu_2_O with empty {100} facets (type I nanoframes), {110} facets (type II nanoframes) and nanocages. Reproduced with permission.^114^ Copyright 2008, American Chemical Society. b) High‐magnification SEM image of a Cu_2_O nanoframe with empty {100} faces. Reproduced with permission.^42^ c) The preferential adsorption between ethanol molecules and *o*‐Cu_2_O crystals: apexes (pink) > edges (yellow) > facets (orange/pea green/black)”. Reproduced with permission.^130^ Copyright 2011, Royal Society of Chemistry. d) SEM images of Cu_2_O jagged polyhedrons. Reproduced with permission.^44^ e) The morphological evolution of uniform Cu_2_O NCs in a weak acetic acid solution. Reproduced with permission.^132^ Copyright 2011, American Chemical Society.

CA adsorption is conducive to the selective etching on different positions of Cu_2_O NCs. M. H. Huang et al.^114^ obtained Cu_2_O nanocages and nanoframes with empty {100} or {110} facets from Cu_2_O truncated rhombic dodecahedra. Because SDS selectively adsorbed and protected the {110} facets, the etching process occurred prior to the {100} facets. Thus, truncated rhombic dodecahedral Cu_2_O nanoframes consisting of empty {100} facets and {110} skeleton facets (type‐I nanoframe) were formed first (**Figure** **7**a). Then, {100} facets were filled during the further reaction, generating nanocages. By adding ethanol and subsequent sonication of the reaction system, the adsorbed SDS on {110} facets of the nanocages was detached which conduced preferential etching of the {110} facets via HCl, leading to the generation of elliptical pores on {110} facets (type‐II nanoframes in Figure 7a).

In order to obtain more delicate structures, a “pre‐synthesis strategy” has been widely used to carve NCs involving a two‐stage route where NCs acted as precursors for subsequent etching.^41^, ^42^, ^46^, ^150^, ^151^ H. B. Yang et al.^42^ reported other Cu_2_O nanocages and nanoframes with empty {100} facets from truncated octahedral Cu_2_O precursors (Figure 7b). The capping PVP preferentially adsorbed onto the {111} facets of the Cu_2_O polyhedra and “freezes” the {111} planes; thus, the subsequent oxidative etching selectively occurred on the {100} facets. Similarly, S. D. Sun et al.^130^ reported the branching growth of Cu_2_O NCs via selective oxidative etching with ethanol solution (Figure 7c). As for *o*‐Cu_2_O, the adsorption energies (*E*) are in the following order: *E*_apex_ > *E*_edge_ > *E*_facet_, according to the different numbers of coordinated O atoms. Hence, the relative order of ethanol molecules adsorbed on *o*‐Cu_2_O should be facet < edge < apex. Therefore, the selective oxidative etching was reversed to the order of adsorption, from the centre of {111} facets, edges, to apexes.

Without CAs, the {111} or {110} facets with higher surface energy are etched prior to the {100} facet. Q. Hua et al.^131^ reported the facet‐dependent oxidative dissolution of *c*‐Cu_2_O, *o*‐Cu_2_O and *d*‐Cu_2_O NCs in NH_3_ solution. The relative stability of different Cu_2_O crystal facets in NH_3_ solution in the sequence of {110} < {111} < {100} that were reversed to the order of the surfaces energies. When changing to a weak acid solution^132^ instead of the aqueous ammonia, the stability of Cu_2_O facets also followed the order of {100} > {111} > {110}, which determined the extent of oxidative dissolution. Stable {100} facets were preserved, but unstable {110} and {111} facets were etched with newly formed {100} facets (Figure 7e). Using truncated octahedron Cu_2_O NCs exposed with {100} and {111} facets as precursors, our group^44^ created Cu_2_O jagged polyhedra totally enclosed by {100} facets, with numerous {111} corners and {110} edges (Figure 7d). Due to the Cu dangling bonds, O_2_ molecules strongly interacted with the {111} facet, making those facets easily dissolute. The selective oxidative etching only occurred on the {111} facets. New {100} facets emerged from the {111} facets by etching, while the original {100} facets remained unchanged.

## 5 Sacrificial Templates

**Figure 8:**
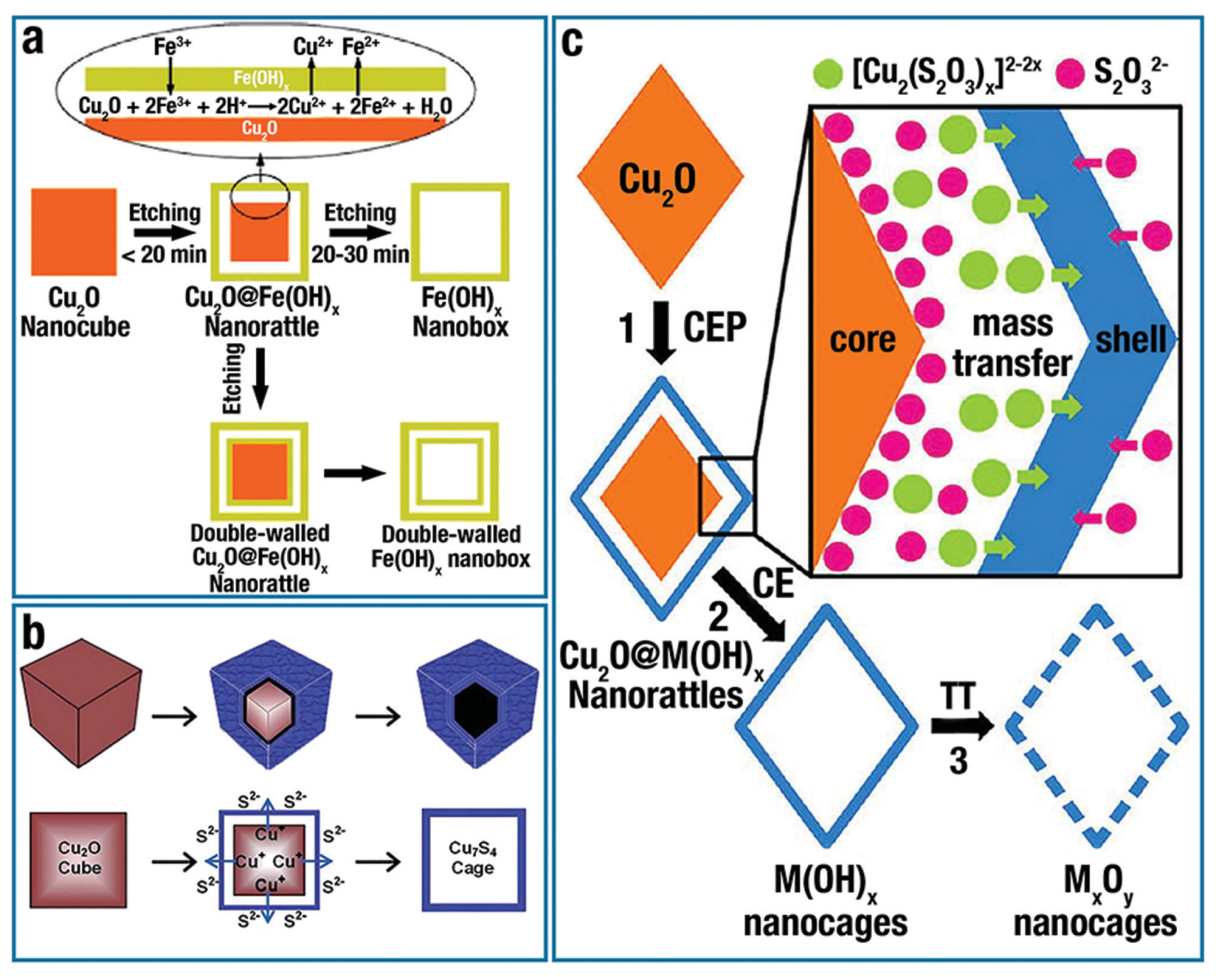
Growth schematic of a) single‐walled or double‐walled Fe(OH)*_x_* by galvanic replacement. Reproduced with permission.^49^ Copyright 2010, American Chemical Society. b) Cu_7_S_4_ hollow nanocubic structure by Kirkendall effect. Reproduced with permission.^158^ Copyright 2014, Royal Society of Chemistry. c) M(OH)*_x_* (M = Mn, Fe, Co, Ni, and Zn) nanocages by coordinating etching of Cu_2_O NCs, and the production of M*_x_*O*_y_* by thermal treating of relevant M(OH)*_x_*. Abbreviations: CEP, coordinating etching and precipitating; CE, coordinating etching; TT, thermal treatment. Reproduced with permission.^47^ Copyright 2013, American Chemical Society.

Due to the large surface area, low density, good surface permeability, and high loading capacity, the shape‐controllable synthesis of hollow/cage‐like nanostructures, even with non‐spherical shapes and regular interiors, has received extensive attention in recent years because of their widespread applications.^38^, ^47^, ^156^ A template‐assisted synthetic strategy is straightforward for the preparation of nanocages and the possible creation of nonspherical nanostructures.^47^, ^49^, ^157^ The following steps occur during the template synthesis of cage‐like/hollow nanostructures: i) synthesizing template, ii) using template to create target structure, iii) removing template (if necessary).^157^ Recently, one “top‐down” synthetic route has been extensively studied by using the low‐cost and highly chemically reactive Cu_2_O NCs (cubes, octahedra, and other highly symmetrical structures) as the sacrificial template, to create various hollow, non‐spherical nanostructures, including hollow metal oxides,^35^, ^38^, ^45^, ^47^, ^48^, ^49^, ^52^ hollow copper sulfide (Cu*_x_*S*_y_*),^93^, ^158^, ^159^, ^160^, ^161^, ^162^ and hollow metals or alloys.^134^, ^163^, ^164^, ^165^ In this section, we summarize the recent progress in Cu_2_O sacrificial templates, and discuss the three major routes as shown in **Figure** **8** (galvanic replacement, the Kirkendall effect, and coordinating etching).

### 5.1 Galvanic Replacement

Galvanic replacement is an electro‐chemical process, in which the sacrificial template is oxidized and dissolved in the solution; meanwhile another metal ion with a higher reduction potential would be reduced and deposited on the surface of the template, and finally inherits the original structure.^39^ For example, due to the lower standard reduction potential of Cu^2+^/Cu_2_O (0.203 V vs standard hydrogen electrode (SHE)) than that of the Fe^3+^/Fe^2+^ pair (0.77 V vs SHE), Fe(III) ions could instantly oxidize a Cu_2_O template at room temperature. This redox reaction is showed in Eq. (2), 49: (2)
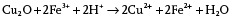



**Figure 9:**
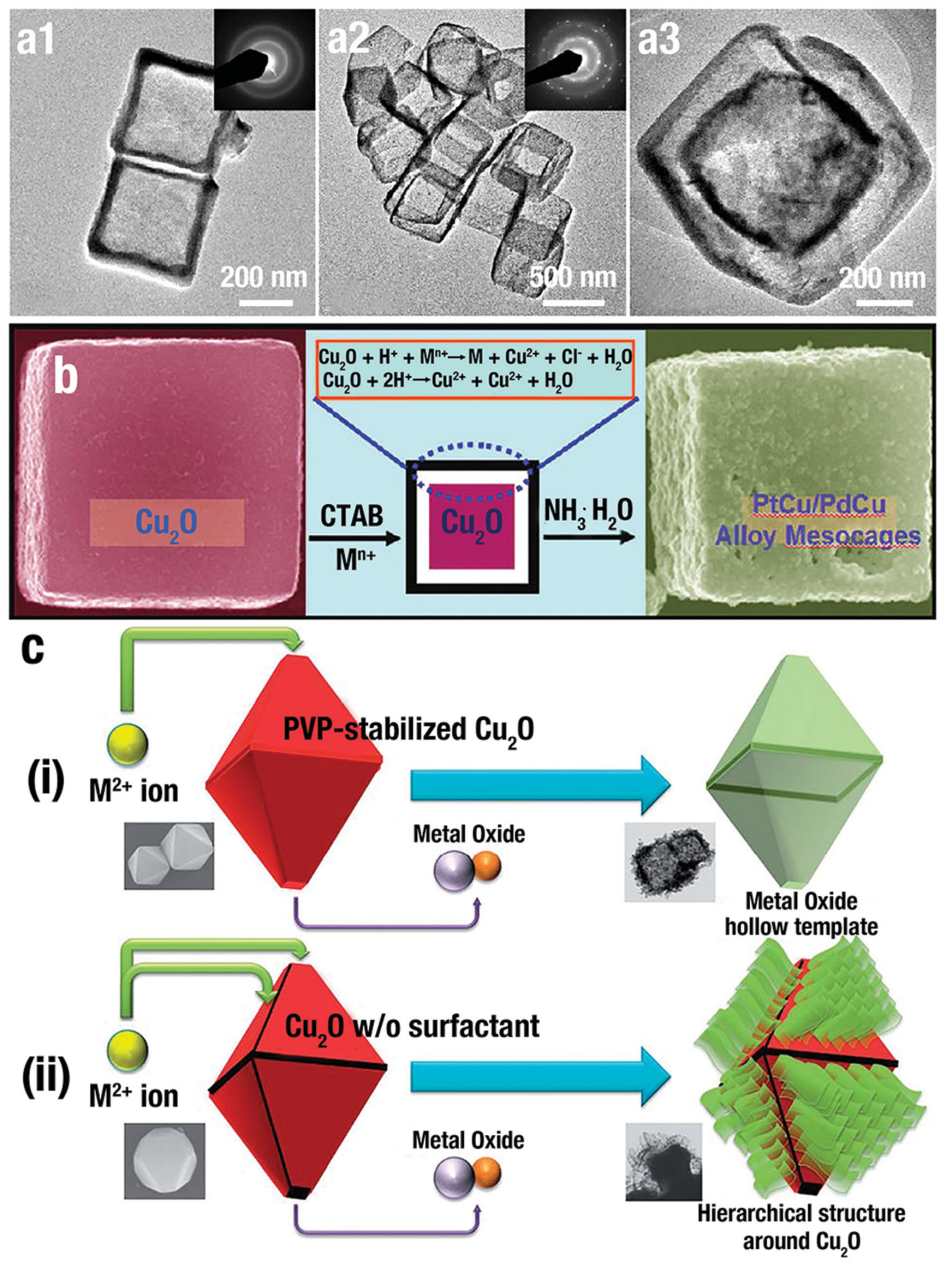
TEM images of a1) Fe(OH)*_x_* nanoboxes, a2) *α*‐Fe_2_O_3_ nanoboxes and a3) Fe(OH)*_x_* box‐in‐box structures. Inset of (a1) and (a2) is the corresponding SAED pattern. Reproduced with permission.^49^ Copyright 2010, American Chemical Society. b) Schematic illustration of the formation of noble metal alloy mesocages from *c*‐Cu_2_ O. Reproduced with permission.^134^ Copyright 2011, American Chemical Society. c) Schematic presentation of the i) PVP‐Cu_2_O and ii) non‐PVP‐Cu_2_O etching reaction behaviour by metal(II) ions. Reproduced with permission.^166^ Copyright 2013, Royal Society of Chemistry.

Amorphous Fe(OH)*_x_* nanoboxes (**Figure** **9**a1) with thin and smooth shells perfectly duplicated the shape of *c‐*Cu_2_O templates. After an annealing process, polycrystalline *α*‐Fe_2_O_3_ nanoboxes were obtained (Figure 9a2). Fe(OH)*_x_* box‐in‐box structures could be created through further redox etching of the Cu_2_O/Fe(OH)*_x_* core/shell (Figure 9a3). Due to the higher standard reduction potential of Pd^2+^/Pd (0.987 V vs SHE) and PtCl_6_^2−^/Pt (0.735 V vs SHE) pairs, Cu_2_O polyhedra could also use for the preparation of nonsperical metal mesocages. F. Hong et al.^134^ synthesized noble metal alloy mesocages (Pd, Pt and Pt/Pd) with many morphologies (cube, octahedron, “star”). Figure 9b illustrates the generation process of metal mesocages from *c*‐Cu_2_O.

Galvanic replacement has facet selectivity when the surface of template possesses more than one type of facet.^139^ Similar to the etching process, galvanic replacement also begins with the facet(s) with the highest surface energy. Certainly, the surface energies of facets can also be altered and even reverse their order via the adsorption of CA.^39^ Using PVP to stabilize the {111} facets of Cu_2_O truncated octahedra, Y. S. Kang et al.^166^ obtained metal oxide hollow structures by controlling the galvanic replacement occurring on the {100} facets (Figure 9ci). In contrast, galvanic replacement and subsequent selective deposition would happen on the {111} facet of Cu_2_O crystals without the protection of PVP, leading to the formation of hierarchical structures (Figure 9cii).

### 5.2 The Kirkendall Effect

The Kirkendall effect is defined as the migration of the boundary layer between two materials when the two materials have different interdiffusion rates. Due to the faster diffusion rate, voids would be formed in the inner component, which is the most defining feature of the Kirkendall effect.^39^ Over the past decade, the Kirkendall effect has become a promising route for creating micro–nano materials with hollow structures.^167^, ^168^ Compared to the mono‐stoichiometric Cu_2_O, copper sulfides (Cu*_x_*S) at room temperature possess at least five stable phases: i.e., chalcocite (Cu_2_S), djurlite (Cu_1.95_S), digenite (Cu_1.8_S), anilite (Cu_1.75_S), and covellite (CuS).^93^ Their unique electrical and optical properties derive from the valence states and complicated structures.^93^, ^96^ The Cu_2_O‐template route (Figure 8b) is a facile and straightforward by adding sulfur sources (i.e., Na_2_S solution, thioacetamide, and thiourea) into the Cu_2_O suspension, in which Cu_2_O template is transformed into Cu_2_O/Cu*_x_*S core/shell structures at once because of the minimal solubility product constant *K*_sp_ of Cu*_x_*S (*K*_sp_ ≈ 10^−48^).^93^ Finally, the Cu_2_O core is dissolved completely, and the Cu*_x_*S shell is kept to the formation of hollow structures.

**Figure 10:**
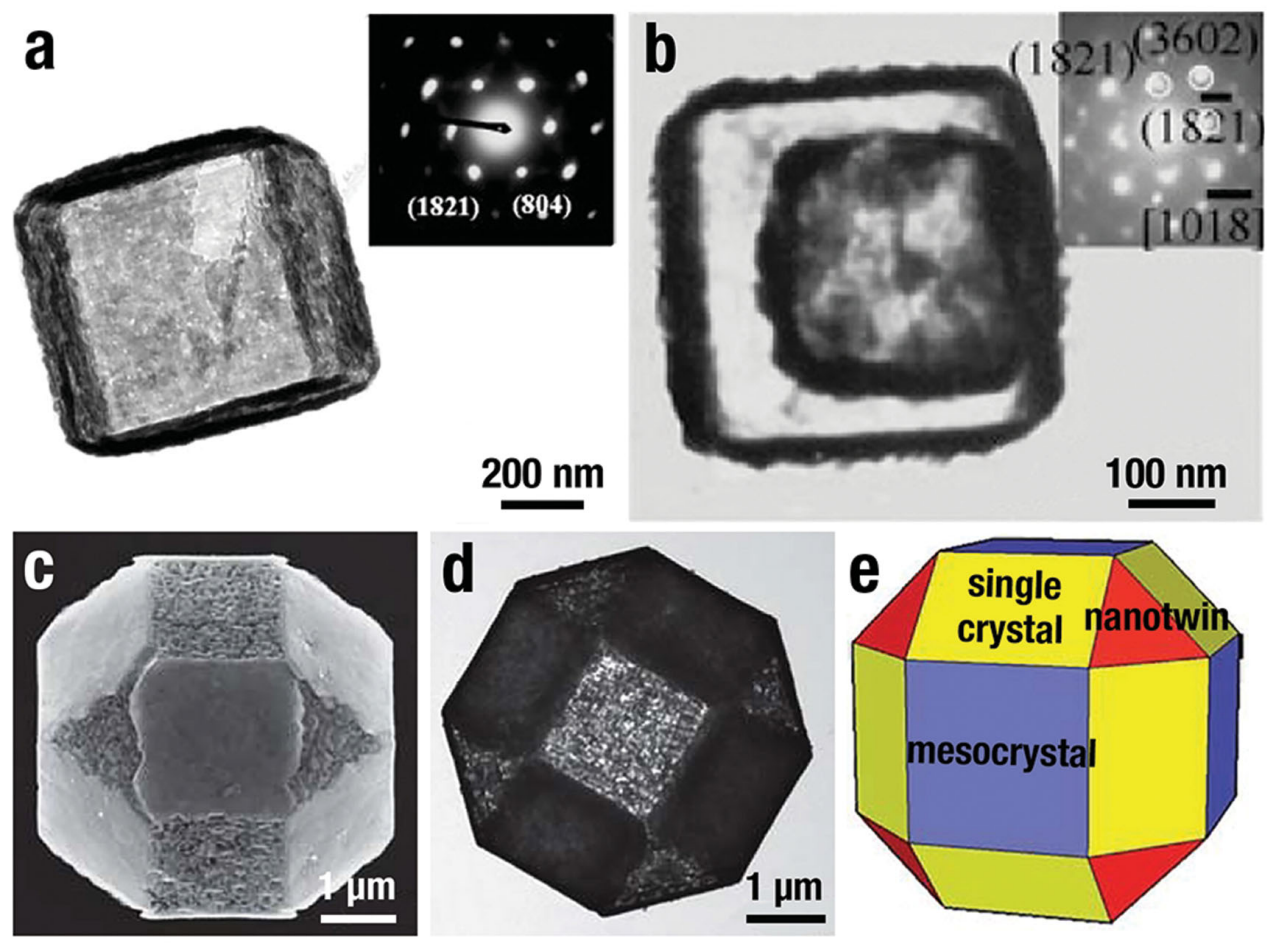
a) TEM image of Cu*_x_*S cages. Inset of (a) is the SAED pattern of the Cu*_x_*S cage. Reproduced with permission.^93^ b) TEM images of double‐walled Cu_7_S_4_ nanoboxes. Inset of (b) is the SAED pattern of the single nanobox. Reproduced with permission.^160^ Copyright 2009, Royal Society of Chemistry. c,d) SEM and TEM images of a individual 26‐facet Cu_7_S_4_ microcage, and e) the corresponding simulated structure. Reproduced with permission.^161^ Copyright 2011, Royal Society of Chemistry.

By using Cu_2_O crystals as templates, D. S. Xu et al.^93^ first created non‐spherical Cu*_x_*S mesocages (including cubic, octahedral and multi‐pod) with single‐crystalline shells. **Figure** **10**a is a typical TEM image of cubic Cu*_x_*S cages. Through a replacement reaction between S^2−^ in solution and O in the Cu_2_O lattices, Cu_2_O/Cu*_x_*S core/shell structures were firstly formed; Cu*_x_*S mesocages obtained through a subsequently removing the residual Cu_2_O by ammonia. It is noteworthy that the compositions can be adjusted from Cu_2_S to Cu_1.75_S through controlling the reaction atmospheres from N_2_ to air as shown in Eq. (3) and Eq. (4), respectively. (3)
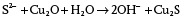

(4)
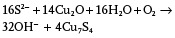



Guided by the above mechanisms, W. X. Zhang et al.^160^ synthesized double‐walled Cu_7_S_4_ nanoboxes by two consecutive cycles that repeatedly produced Cu_7_S_4_ layers in Na_2_S solution and dissolved the Cu_2_O core in NH_3_ solution (Figure 10b). Using polyhedral 26‐facet Cu_2_O microcrystals as the templates, S. D. Sun et al.^161^ synthesized 26‐facet CuS microcages with different types of shells (Figure 10c–e). Interestingly, the three pairs of square {100} facets and the four pairs of triangular {111} facets became rough with self‐assembled nanoplate, however, the six pairs of rectangular {110} facets remained smooth. Further TEM analysis demonstrated that the {100}, {111} and {110} facets of CuS microcage were transformed into mesocrystal, nano‐twin and single crystal, respectively colored blue, red, and yellow in the simulated structure shown in Figure 10e. The formation of different shells is attributed to the different crystallographic structures of {100}, {111} and {110} facets of Cu_2_O crystals. The {110} and {111} facets with dangling Cu atoms could be protected by negatively charged agents, while the neutral {100} facet had weak protection. Thus, the rate of the Kirkendall process between S and O atoms was different in each surface, leading to different shells.

### 5.3 Coordinating Eetching

Coordinating dissolution is commonly used for dissolving insoluble materials. For instance, by using certain ligands (CN^−^, SCN^−^, S_2_O_3_^2−^, Cl^−^ or NH_3_ etc.) coordinate Cu_2_O polyhedra, various transition metal hydroxides, or oxides with hollow structures could be obtained, perfectly imitating the geometry of the Cu_2_O template. Z. Y. Wang et al.^48^ synthesized uniform SnO_2_ nanoboxes by combining precisely controlled hydrolysis of SnCl_4_ and simultaneous coordinating etching of the *c*‐Cu_2_O templates. During this process, insoluble CuCl intermediate was immediately formed, and dissolved in NaCl solution via coordinating with excess Cl^−^ to form soluble [CuCl*_x_*]^1−*x*^. Eventually, outward evacuation of [CuCl*_x_*]^1−*x*^ and inward diffusion of Sn^4+^ and Cl^−^ through the SnO_2_ shell lead to formation of intact SnO_2_ shells and the consumption of Cu_2_O templates. Based on the Pearson's hard and soft acid–base (HSAB) principle, stable complexes could be formed through the interaction of hard bases with hard acids, and soft bases with soft acids. As a soft acid, Cu^+^ within the Cu_2_O templates prefer a soft base ligand (S_2_O_3_^2−^, CN^−^, SCN^−^) to a hard base (Cl^−^, NH_3_) as the coordinating etchant. Recently, our group^47^ put forward a general route to create metal hydroxides (MHs, M = Mn, Fe, Co, Ni, and Zn) nanocages by employing *o‐*Cu_2_O as the sacrificial template at room temperature. (5)
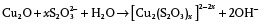

(6)
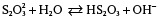

(7)
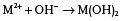



The strategy was well designed by using Na_2_S_2_O_3_ as the coordinating etchant. S_2_O_3_^2−^ would coordinate etching Cu_2_O and form soluble [Cu_2_(S_2_O_3_)*_x_*]^2−2*x*^, because the soft−hard interaction of Cu^+^−O^2−^ within Cu_2_O was weakened compared to the soft−soft interaction of Cu^+^−S_2_O_3_^2−^ (Eq. (5)). Due to the unstable interaction of borderline acid−soft base (M^2+^ − S_2_O_3_^2−^), metal ions (M^2+^) were free in the solution. The OH^−^ ions that originated from Cu_2_O etching (Eq. (5)) and some S_2_O_3_^2−^ hydrolysis (Eq. (6)) lead to the generation of M(OH)_2_ (Eq. (7)). The above routes could be concluded as “coordinating etching and precipitating”, which is shown as Step 1 in Figure 8c. The two simultaneous reactions ensure that the shell of M(OH)_2_ perfectly kept the original shape of *o*‐Cu_2_O (Step 2 in Figure 8c). Through simple thermal treatment, those polyhedral amorphous MHs nanocages could dehydrate into polycrystalline metal oxide (MO) porous nanocages (Step 3 in Figure 8c).

**Figure 11:**
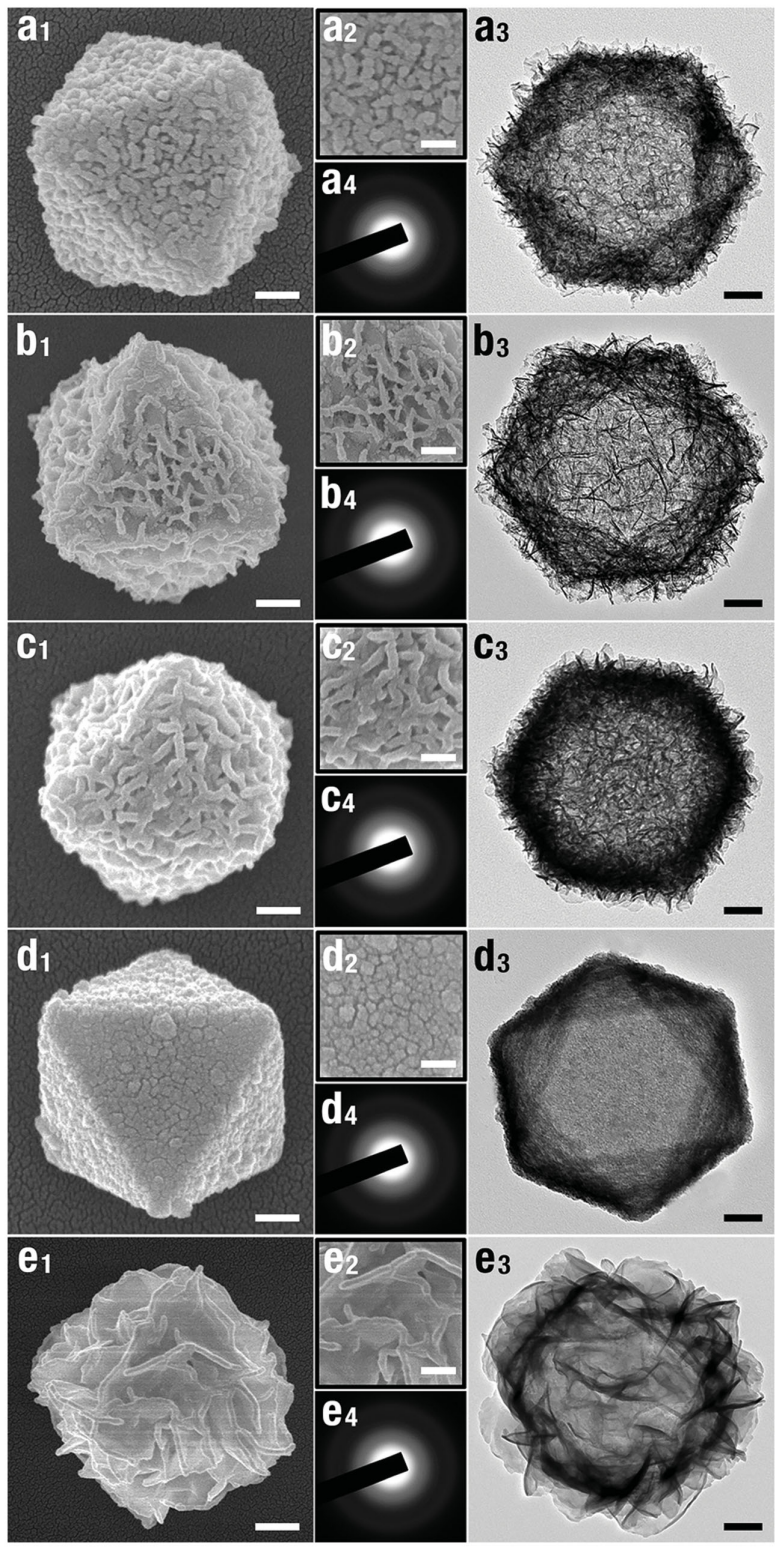
SEM, TEM, and SAED images of the a) Mn(OH)_2_, b) Fe(OH)_2_, c) Co(OH)_2_, d) Ni(OH)_2_, and e) Zn(OH)_2_ nanocages. Parts (*x*_1_)(*x* = a–e) and (*x*_3_) display typical SEM and TEM images of MH nanocages, respectively; part (*x*_2_) displays high‐magnification SEM images of part (*x*_1_); part (*x*_4_) is the corresponding SAED patterns. The scale bars in parts (*x*_1_), (*x*_2_), and (*x*_3_) are 100, 20, and 100 nm, respectively. Reproduced with permission.^47^ Copyright 2013, American Chemical Society.

**Figure 12:**
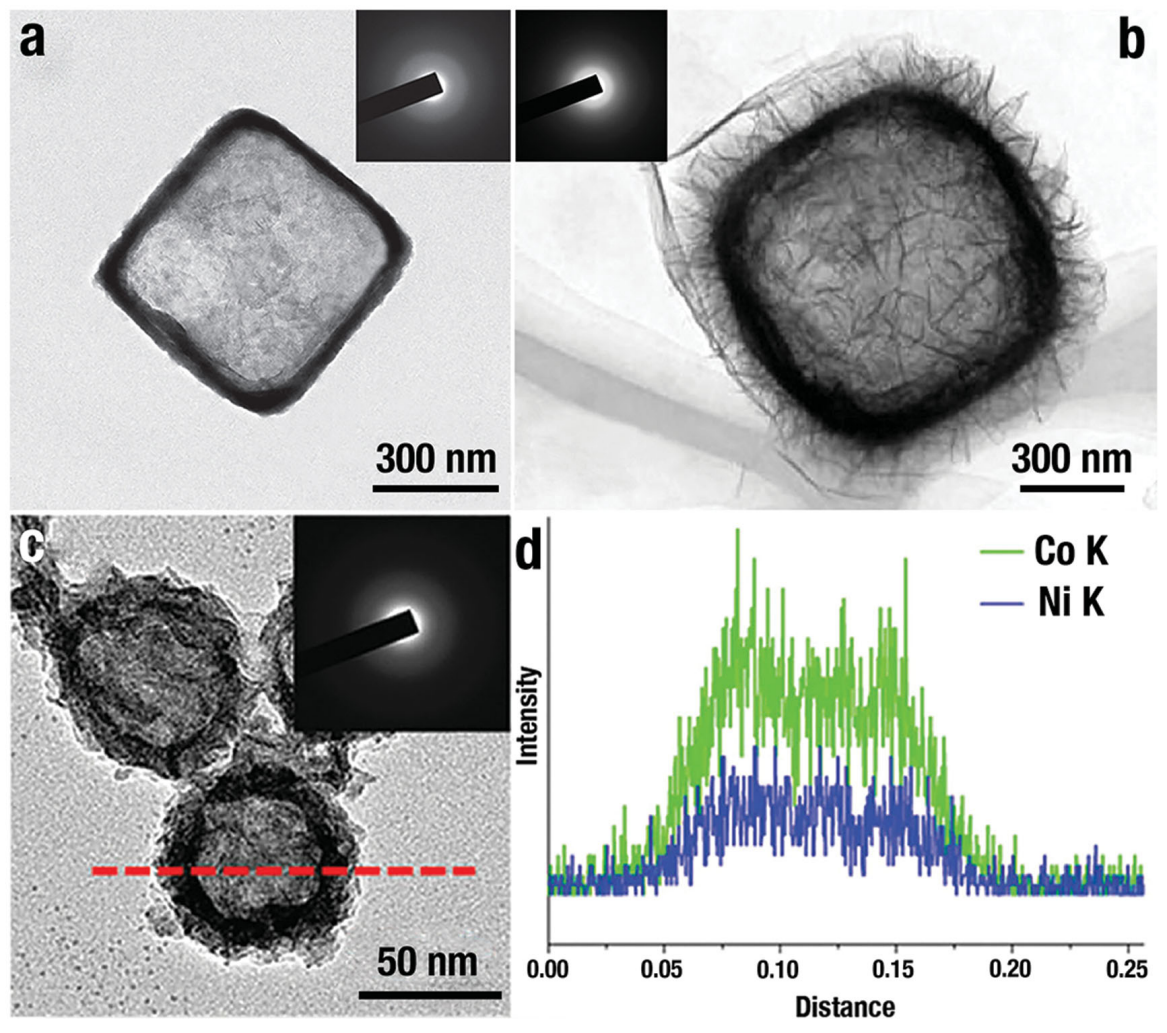
a) Typical TEM image of Ni(OH)_2_ nanobox with thin shell and well‐defined interior. Reproduced with permission.^52^ b) Typical TEM image of Co(OH)_2_/rGO with secondary structures. Reproduced with permission.^45^ Copyright 2014, American Chemical Society. c) A typical TEM image of NiCo_2.7_(OH)*_x_* double hydroxides nanocages, and d) their corresponding EDS measurements. Reproduced with permission.^35^ Inset of a–c) is the SAED pattern of each hydroxides nanocages.

Well‐defined MH nanocages (Mn(OH)_2_, Fe(OH)_2_, Co(OH)_2_, Ni(OH)_2_, and Zn(OH)_2_) could be produced according the CEP route (**Figure** **11**). The as‐prepared MH nanocages kept the shape of the *o*‐Cu_2_O template with an edge length of ≈500 nm (Figure 11 *x*_1_; *x* = a–e), and small NPs consisted of the MH shell (Figure 11 *x*_2_). TEM images of MH nanocages (Figure 11 *x*_3_) clearly illustrated their hollow characteristic, and the SAED patterns (Figure 11 *x*_4_) demonstrated their amorphous in nature. According to this strategy, amorphous Ni(OH)_2_ nanoboxes^52^ (**Figure** **12**a), Co(OH)_2_/reduced graphene oxide^45^ (Figure 12b) and Ni–Co amorphous double hydroxides^35^ (Figure 12c,d) can also be obtained by minor revised this method, and illustrate excellent performances in the realm of sensor and energy.

## 6 Applications in Catalysis and Sensing

To date, the applications of Cu_2_O have mainly been in the realms of catalysis and sensing. In this section, we will focus on the facet‐dependent performances of the three basic Cu_2_O NCs and such Cu_2_O‐based NCs for applications of photocatalysis, gas catalysis, organocatalysis, and sensing as well as the relationship between their structures and properties.

### 6.1 Photocatalysis

**Figure 13:**
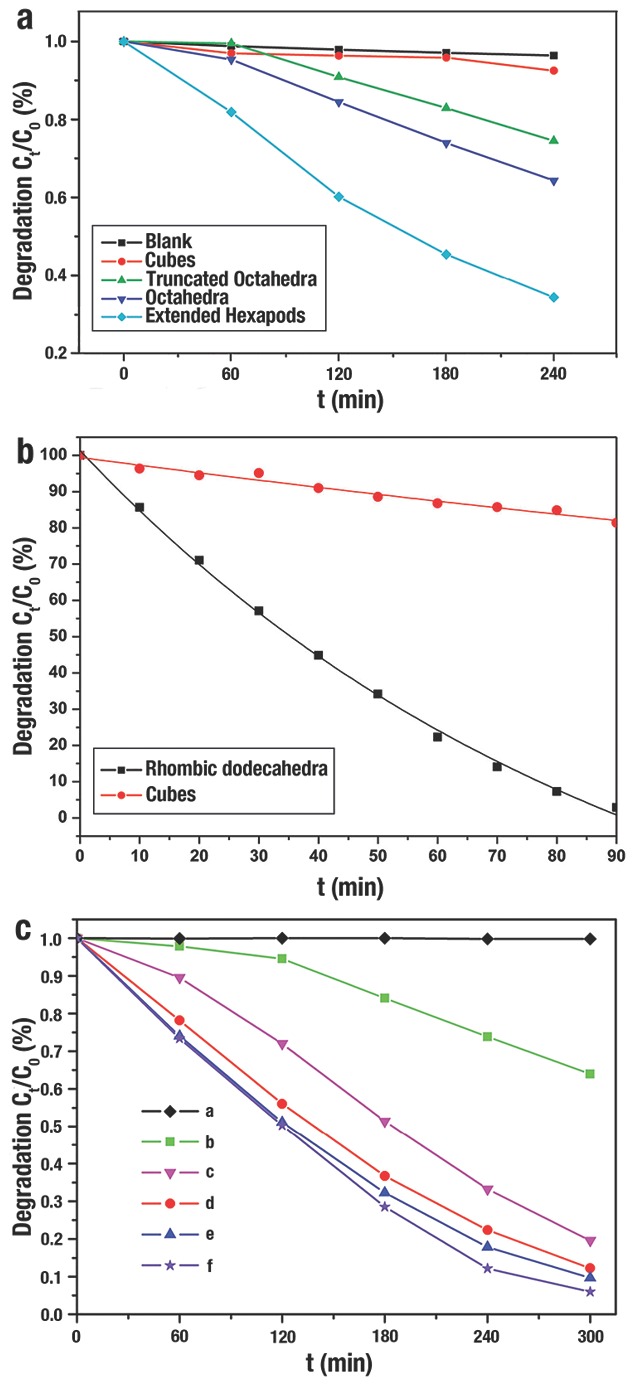
a) Degree of photodecomposition of MO vs time by using different Cu_2_O NCs as the photocatalysts. The blank sample only contain the MO solution without Cu_2_O NCs. Reproduced with permission.^128^ Copyright 2009, American Chemical Society. b) Degree of photodecomposition of MO vs time by using *d*‐Cu_2_O and *c*‐Cu_2_O as the photocatalysts. Reproduced with permission.^75^ Copyright 2012, American Chemical Society. c) Degree of photodecomposition of MO vs time by using different Cu_2_O Cu_2_O NCs as the photocatalysts: (a) blank sample; (b) *c*‐Cu_2_O; (c) *o*‐Cu_2_O; (d) 50‐facet polyhedra with {211}; (e) 50‐facet polyhedra with {522} and (f) 50‐facet polyhedra with {311}. Reproduced with permission.^135^ Copyright 2012, Royal Society of Chemistry.

The requirement of sustainable energy and reduction of environmental pollution has driven considerable research efforts in photo‐degradation of pollutants and water splitting by employing abundant solar energy.^169^ Cu_2_O with bandgap of 2.1 eV are expected to be promising materials in visible‐light photocatalytic degradation,^69^ and great studies have been devoted to the controlled synthesis of Cu_2_O with their morphology‐dependent photocatalytic activities.^33^, ^36^, ^44^, ^73^, ^114^, ^147^ During the photocatalytic process, one of the key factors for the catalyst is “catching” the organic pollutants, since that would offer the catalyst more opportunities to contact and catalyze those pollutants.^44^, ^75^ Our group^7^ demonstrated that the adsorption ability of methyl orange (MO), one of the industrial pollutants, to the different shapes of Cu_2_O NCs followed the sequence of octahedra > cubooctahedra > cubes. The exposed {111} facets of *o*‐Cu_2_O had positively charged “Cu” atoms that inclined to interact with the negatively charged groups –SO_3_^−^ in MO molecules. This suggested that Cu_2_O {111} facets would strongly interact with the molecules possessing negatively charged groups, and then effectively photodecompose these molecules; while the {111} facets interact weakly with the positively charged molecules, and lead to a poor photodegradation activities. As expected, M. H. Huang et al.^128^ verified that the photocatalytic activity of *o*‐Cu_2_O was higher than that of *c*‐Cu_2_O. Furthermore, the photocatalytic activities of extended hexapods Cu_2_O NCs with more {111} facets were more effective and active than *o*‐Cu_2_O (**Figure** **13**a). Subsequently, they synthesized *d*‐Cu_2_O NCs with only exposed {110} facets,^75^ which exhibited an excellent photocatalytic activities for the photodegradation of MO because of the high density of Cu atoms on the surface (Figure 13b). T. R. Zhang et al.^135^ demonstrated the photocatalytic activities of Cu_2_O microcrystals: *c*‐Cu_2_O with {100} facet <*o*‐Cu_2_O with {111} facet < 50‐facet polyhedral with {211} facet ≈ 50‐facet polyhedra with {522} facet ≈ 50‐facet polyhedral with {311} facet (Figure 13c). The larger number of atomic steps and kinks in these high‐index facets contributed to the more efficient photodegradation than those of the *c*‐Cu_2_O and *o*‐Cu_2_O.

**Figure 14:**
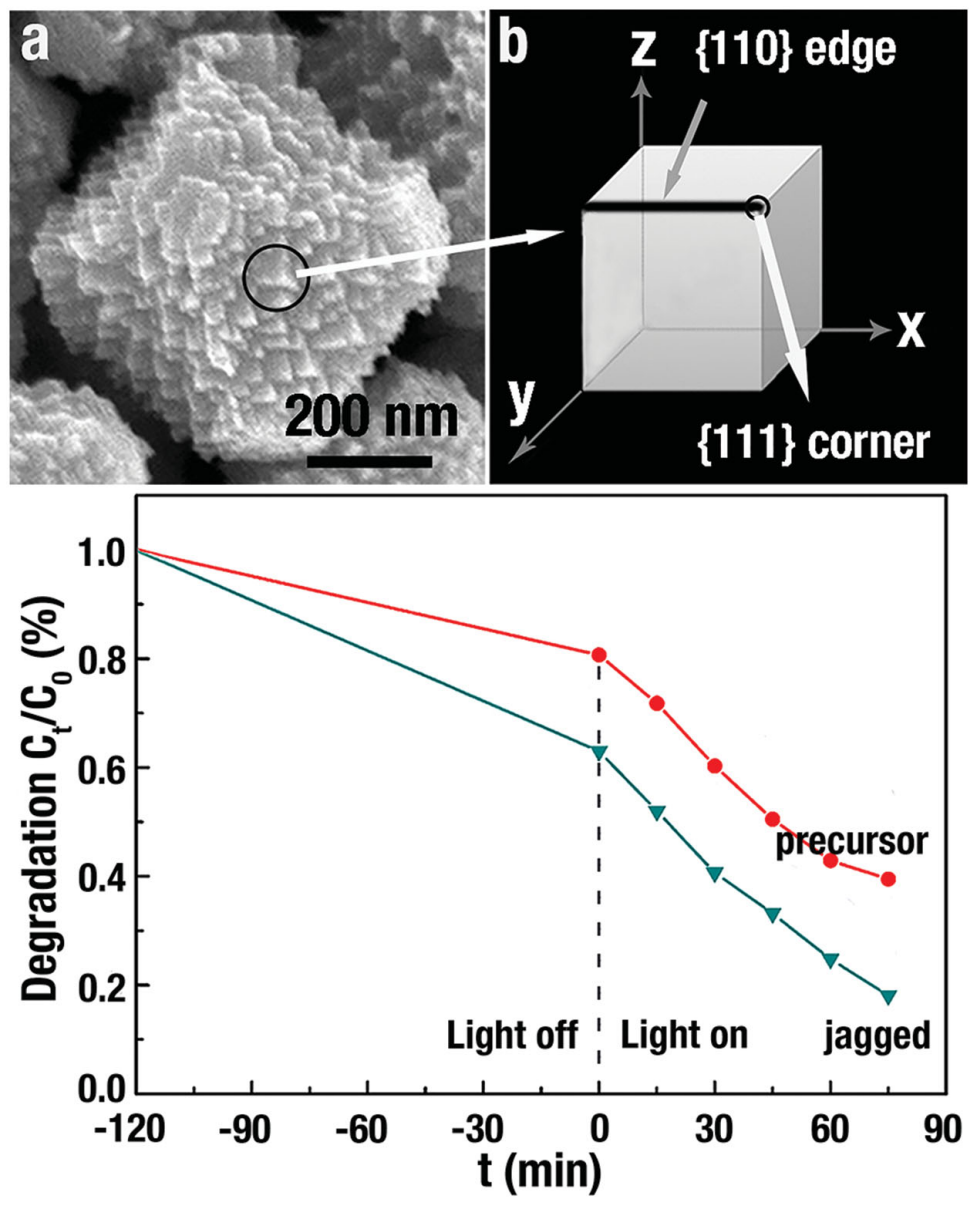
a,b) SEM image and illustration of the triangular pyramids of Cu_2_O jagged polyhedron. c) Degree of photodecomposition of MO vs time by using Cu_2_O precursor and jagged polyhedron as the photocatalysts. Reproduced with permission.^44^

A more efficient photogenerated electron–hole (e^−^/h^+^) pair separation would contribute to the improvement of photocatalytic activity. Besides the strong interaction between MO and the {111} corners and {110} edges of Cu_2_O jagged polyhedron (**Figure** **14**a,b), the OH^−^ ions also selectively adsorb onto these corners and edges with higher energy. Thus, a faster e^−^/h^+^ separation will accelerate the production of the ·OH free radicals and then enhance their photocatalytic activities. Compared to the precursor of Cu_2_O truncated octahedron, the Cu_2_O jagged polyhedron displayed a better photocatalytic performance in the degradation of MO (Figure 14c).^44^ After 75 min, MO was only degraded to 60% by the Cu_2_O precursor, while MO was even degraded to 82% by jagged Cu_2_O.

**Figure 15:**
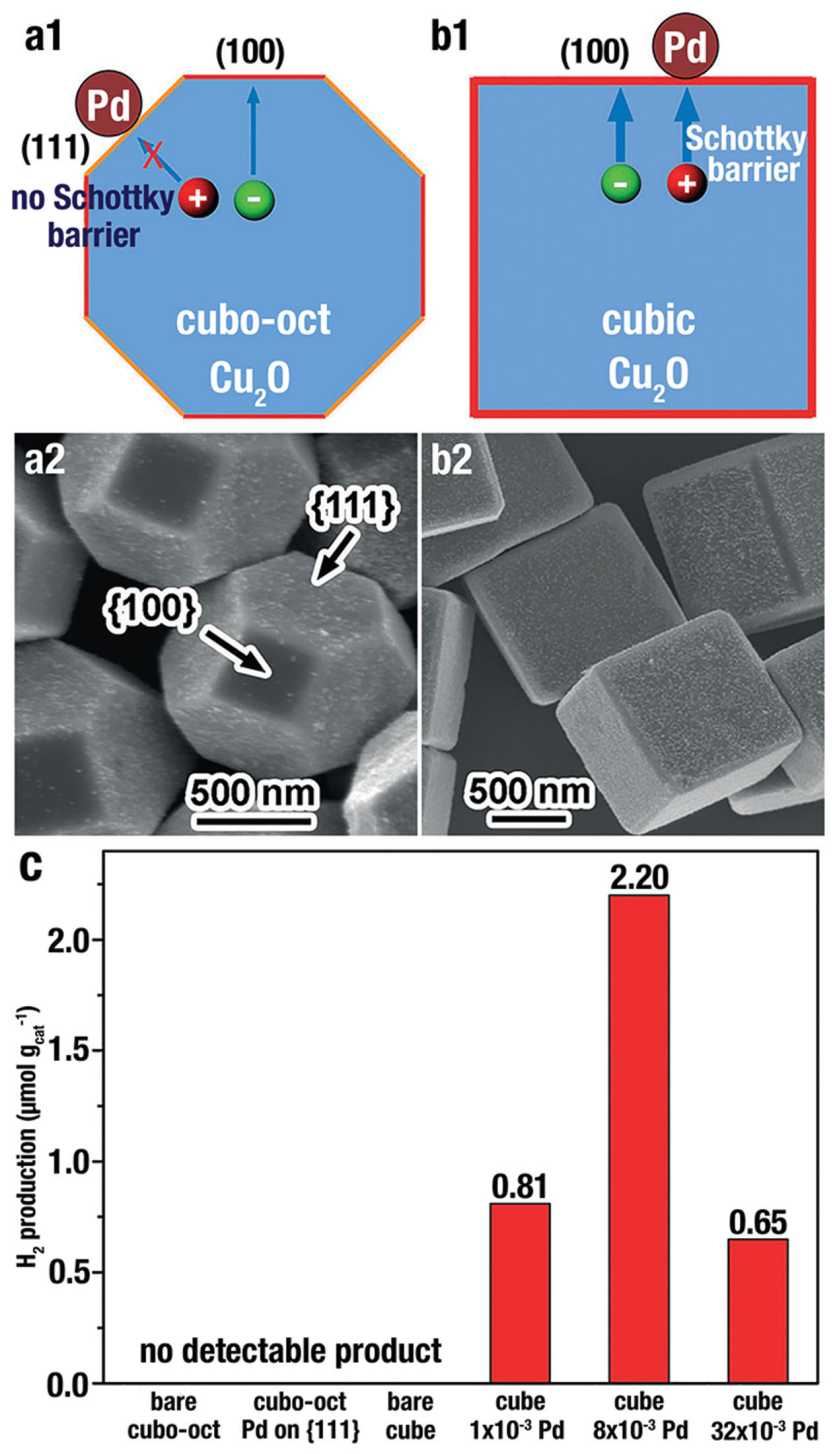
a1,b1) Scheme of the photogenerated charge transfer in the Pd‐Cu_2_O cubo‐octahedron and Pd‐Cu_2_O cubes, respectively. a2,b2) SEM image of Cu_2_O cubo‐octahedron and Cu_2_O cubes with Pd selectively loaded on the {111} and {100} surface, respectively. The molar ratio of Pd/Cu_2_O in b2 is 8 × 10^−3^. c) H_2_ production from pure water irradiation for 4 h by employing various photocatalysts under visible‐light (*λ* > 400 nm). “Cubo‐oct” denotes cubo‐octahedron, and the concentrations represent the molar ratio of Pd/Cu_2_O. Reproduced with permission.^33^

Furthermore, another important factor for photodecomposition reactions is the rapid transportation to the surfaces of photo­generated charges. A Schottky barrier could be formed at the metal–semiconductor interface that reduces the recombination of the photogenerated e^−^/h^+^ pairs, and then improve photocatalytic efficiency.^30^, ^33^, ^36^ Y. J. Xiong et al.^33^ designed a p‐type metal–semiconductor (Pd–Cu_2_O) heterostructure, and demonstrated that the synergistic effect between charge spatial separation and Schottky barrier contributed to the efficient hydrogen production from pure water (**Figure** **15**). Due to the low work function of {111} facet, no Schottky barrier is formed at the Pd–Cu_2_O{111} interface; instead, an anti‐blocking layer would be formed at that interface that increase the recombination of e^−^/h^+^ pairs (Figure 15a). In contrast, since the high work function of {100} facet, e^−^/h^+^ pairs would be well separated at the Cu_2_O{100}‐Pd interface (Figure 15b). The hydrogen production of Pd–Cu_2_O cubes with proper Pd load capacity over 4 h was 2.20 mmol g^−1^, which was dramatically higher than other Cu_2_O counterparts (Figure 15c).

### 6.2 Gas Catalysis

**Figure 16:**
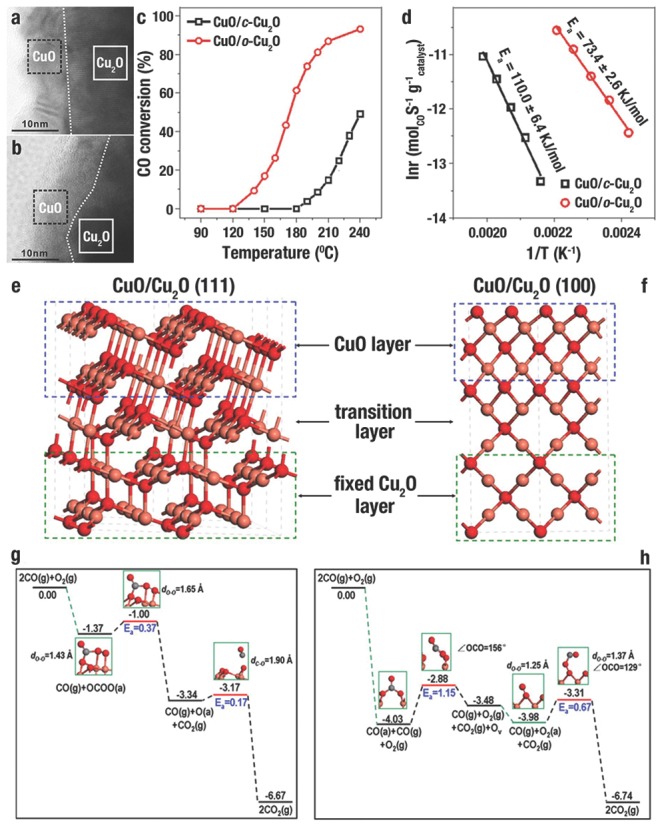
a,b) HRTEM image of CuO thin film formed on Cu_2_O cube (CuO/*c*‐Cu_2_O) and Cu_2_O octahedron (CuO/*o*‐Cu_2_O) during the CO oxidation, respectively. c) Catalytic performance of CuO/*c*‐Cu_2_O and CuO/*o*‐Cu_2_O in CO oxidation. d) The activation energies of CO oxidation catalyzed by CuO/*c*‐Cu_2_O and CuO/*o*‐Cu_2_O showed in the Arrhenius plot. Optimized surface structures of e) CuO/Cu_2_O {111} and f) CuO/Cu_2_O {100}, and the energy profiles of each elementary step in the oxidation of CO catalyzed by g) CuO/Cu_2_O {111} and h) CuO/Cu_2_O {100}. The grey, red and pink spheres denote C, O and Cu atoms, respectively. Reproduced with permission.^2^

Cu_2_O crystals have been actively studied in gas catalysis, and showed facet‐dependent catalytic performance.^2^, ^3^, ^59^, ^170^ W. X. Huang et al.^2^ evaluated the CO oxidation of uniform *c*‐Cu_2_O and *o*‐Cu_2_O. HRTEM images (**Figure** **16**a,b) demonstrated that the surfaces of *c*‐Cu_2_O and *o*‐Cu_2_O were all oxidized to CuO thin films during the CO oxidation (denoted as CuO/*c*‐Cu_2_O and CuO/*o*‐Cu_2_O, respectively). CuO/*c*‐Cu_2_O became active at 190 ºC and achieved a conversion rate of 49.1% CO at 240 ºC, while CuO/*o*‐Cu_2_O became active at 140 ºC and achieved the conversion rate of 93.2% CO at 240 ºC (Figure 16c). The activation energies of CuO/*c*‐Cu_2_O and CuO/*o*‐Cu_2_O oxidized CO were calculated to be 110.0 ± 6.4 and 73.4 ± 2.6 kJ mol^−1^ respectively, as shown in the Arrhenius plot of Figure 16d. Density functional theory (DFT) calculation suggested that CuO thin films grow on {111} and {100} facets with different surface compositions and structures (Figure 16e,f). Three‐coordinated O (O_3c_) and three‐coordinated Cu (Cu_3c_) atoms were terminated at the CuO overlayer on {111} facet (Figure 16e); by contrast, only O_2c_ atoms were terminated at the {100} facet (Figure 16f).

Further DFT calculation results demonstrated that there were significant differences in the reaction process and active sites during the process of CuO/*c*‐Cu_2_O and CuO/*o*‐Cu_2_O oxidized CO (Figure 16g,h). For the surface of CuO/*o*‐Cu_2_O (Figure 16g), individual CO or O_2_ molecules were weakly adsorbed on the sites of Cu_3c_, while these two molecules could be strongly co‐adsorbed on the sites of O_3c_ and Cu_3c_ and then a OCOO(a) surface intermediate (SI) was formed. Subsequently, the OCOO(a) SI disintegrated into CO_2_ and a O(a) adatom, with an activation energy (*E_a_*) of 0.37 eV. Finally, CO reacted with O(a) adatom to produce CO_2_ with a *E_a_* of 0.17 eV; thus, a cycle of catalytic process has completed. For the surface of CuO/*c*‐Cu_2_O (Figure 16h), O_2_ molecules could not adsorb on the sites of O_2c_, while CO could be strongly adsorbed onto the sites of O_2c_ and a CO_3_(a) SI was formed. Next, the CO_3_(a) SI disintegrated to generate CO_2_, and an oxygen vacancy (OV) was created in CuO with a *E_a_* of 1.15 eV. Subsequently, O_2_ molecules could adsorb on OV in CuO to produce O_2_(a). Finally, CO reacted with O_2_(a) to produce CO_2_ with a *E_a_* of 0.67 eV; thus, a cycle of catalytic process has completed, and the OV has refilled. Therefore, DFT calculation results were in accordance with experimental results, which demonstrated that CO oxidation catalyzed by CuO/*o*‐Cu_2_O proceeded with a lower *E_a_* (the disintegration of the OCOO(a), 0.37 eV) than that catalyzed by CuO/*c*‐Cu_2_O (the disintegration of the CO_3_(a) SI, 1.15 eV).

**Figure 17:**
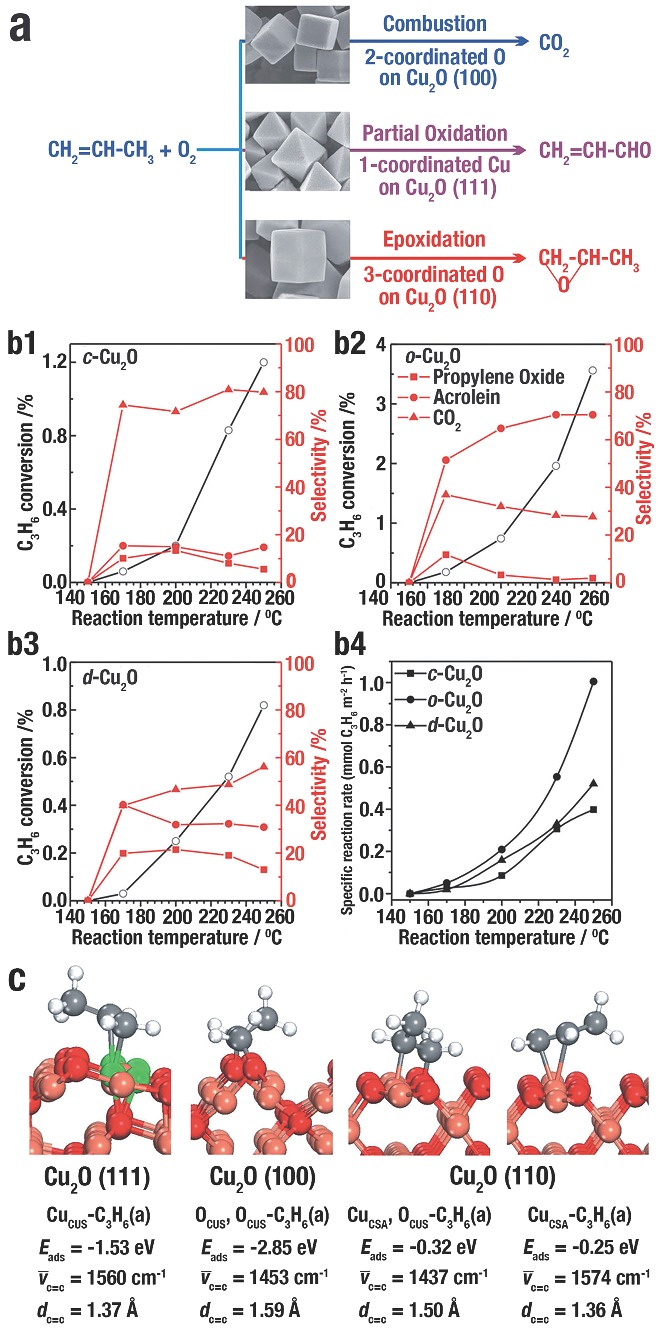
a) Scheme of facet‐dependent selectivity of Cu_2_O catalysed propylene oxidation with O_2_. b1–b3) The conversion of C_3_H_6_ and selectivity of the C_3_H_6_ oxidation with O_2_ for propylene oxide, acrolein, and CO_2_ catalysed by: b1) *c*‐Cu_2_O, b2) *o*‐Cu_2_O, and b3) *d*‐Cu_2_O. b4) Specific reaction rate of the oxidation of C_3_H_6_ with O_2_ catalysed by *c*‐Cu_2_O, *o*‐Cu_2_O, and *d*‐Cu_2_O. c) The most stable structures of C_3_H_6_(a) species on Cu_2_O (111), (100), and (110) facet with the adsorption energy (*E_ads_*), C=C stretching frequency, and distance of C=C bond (*d*_C=C_). White, red, gray, green and pink balls behalf H, O, C, coordinatively unsaturated Cu, and coordinatively saturated Cu, respectively. Reproduced with permission.^3^

W. X. Huang et al.^3^ further reported the facet‐dependent performances in catalyzing propylene oxidation by using CA‐free *c*‐Cu_2_O, *o*‐Cu_2_O, and *d*‐Cu_2_O. *c*‐Cu_2_O enclosed by {100} facets were most selective for CO_2_; *o*‐Cu_2_O exposed {111} facets were most selective for acrolein; *d*‐Cu_2_O enclosed by {110} facets were most selective for propylene oxide (**Figure** **17**a). All the three Cu_2_O NCs became active at 170 ºC, and the conversion rate of C_3_H_6_ rose with the increase of reaction temperature (Figure 17 b1‐b3). In addition, the efficiency of C_3_H_6_ conversion was followed the order *o*‐Cu_2_O > *c*‐Cu_2_O > *d*‐Cu_2_O. The specific reaction rate of propylene for various Cu_2_O NCs also followed the order *o*‐Cu_2_O > *c*‐Cu_2_O > *d*‐Cu_2_O (Figure 17 b4). *o*‐Cu_2_O are more active in catalyzing C_3_H_6_ oxidation with O_2_ than *c*‐Cu_2_O and *d*‐Cu_2_O. The (111) plane was terminated with three‐coordinated unsaturated O (O_CUS_) in the first layer, and one‐coordinated unsaturated Cu (Cu_CUS_) and coordinated saturated (Cu_CSA_ ) in the second layer in a 1:3 ratio. The (100) plane was terminated with two‐coordinated O_CUS_ in the first layer, and Cu_CSA_ in the second layer. The (110) plane was terminated with three‐coordinated O_CUS_ and Cu_CSA_ in the first layer, and Cu_CSA_ in the second layer. DFT calculations explained the adsorption of C_3_H_6_ on (111), (100), and (110) facets (Figure 17b). For the (111) facet (Figure 17c), Cu_CUS_–C_3_H_6_(a) was formed through the selective adsorption of C_3_H_6_ molecules at the site of Cu_CUS_ (C=C stretching frequency (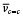) of 1560 cm^−1^) with an adsorption energy (*E_ads_*) of –1.53 eV. For the (100) facet (Figure 17c), O_CUS_,O_CUS_–C_3_H_6_(a) was formed through the selective adsorption of C_3_H_6_ molecules at the site of two neighboring two‐coordinated O_CUS_ (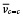 = 1453 cm^−1^) with an *E_ads_* of –2.85 eV. For the (110) facet (Figure 17c), C_3_H_6_ adsorbed on the three‐coordinated O_CUS_, and Cu_CSA_ sites to form Cu_CSA_,O_CUS_–C_3_H_6_(a) (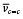 = 1437 cm^−1^), and on O_CUS_ site to generate O_CUS_–C_3_H_6_(a) (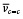 = 1574 cm^−1^). The *E_ads_* for the two sites of (110) facet was similar.

**Figure 18:**
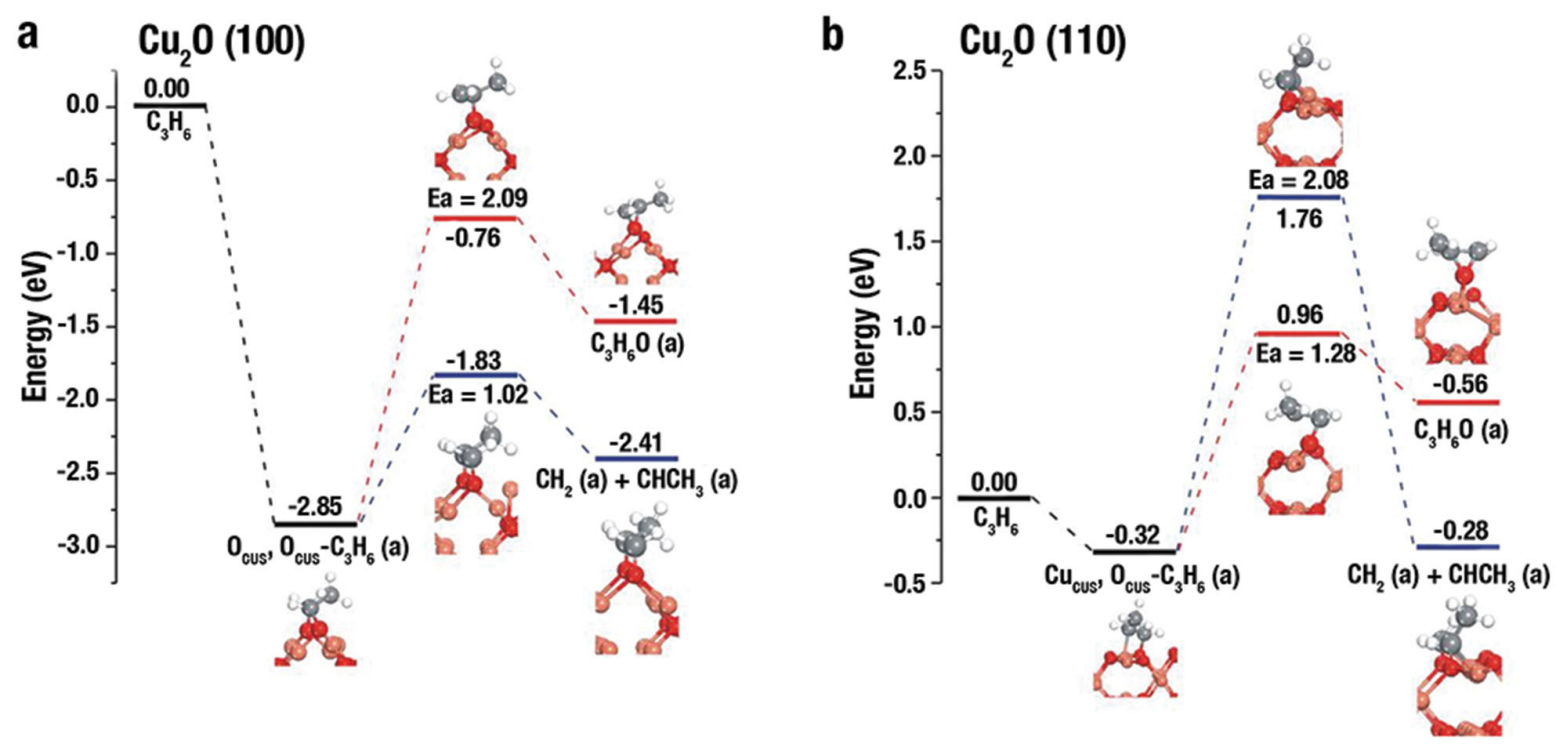
The potential energy surface and corresponding structures for epoxidation and the complete C=C bond breaking of adsorbed propylene on a) Cu_2_O(100) and b) Cu_2_O(110). Pink, red, white, and gray balls represent Cu, O, H, and C atoms, respectively. Reproduced with permission.^3^

In addition, different SI produced on each Cu_2_O NCs. On the Cu_2_O (111) surface, the distance of C=C bond (*d*_C=C_) in Cu_CUS_–C_3_H_6_(a) was calculated to be 1.37 Å, while *d*_C=C_ in C_3_H_6_ molecule was 1.34 Å. Therefore, the stable C=C bond of Cu_CUS_–C_3_H_6_(a) can be kept in the subsequent reactions, which was in favor of the generation of acrolein. On the Cu_2_O(100) surface, d_C=C_ in O_CUS_,O_CUS_–C_3_H_6_(a) was 1.59 Å; hence, the weakened C=C bond would be cleaved by propylene combusting. DFT calculation results (**Figure** **18**a) demonstrated that the *E_a_* for the combustion of O_CUS_, O_CUS_–C_3_H_6_(a) to adsorb C_3_H_6_O(a) and the disintegration of O_CUS_, O_CUS_–C_3_H_6_(a) into adsorbed CH_2_(a) and CHCH_3_(a) was 2.09 and 1.02 eV, respectively. This result suggested that propylene was in favor of combustion, which was in accordance with the experiments of *c*‐Cu_2_O. On the Cu_2_O(110) surface, *d*_C=C_ in O_CUA_–C_3_H_6_(a) was 1.36 Å, which was considered to the generation of acrolein; while the weakened C=C bond in Cu_CSA_,O_CUS_–C_3_H_6_(a) with a distance of 1.50 Å inclined to break during the reactions. DFT calculations results (Figure 18b) suggested that the *E_a_* for the combustion of O_CUA_,O_CUS_–C_3_H_6_(a) to adsorb C_3_H_6_O(a) and the disintegration of O_CUA_,O_CUS_–C_3_H_6_(a) into adsorbed CH_2_(a) and CHCH_3_(a) was 1.28 and 2.08 eV, respectively. This result suggested that propylene was in favor of epoxidation, which was in accordance with the experiments of *d*‐Cu_2_O. The distinction in reactivities between Cu_CSA_,O_CUS_–C_3_H_6_(a) on Cu_2_O(110) and O_CUS_,O_CUS_–C_3_H_6_(a) on Cu_2_O(100) is three‐coordinated O_CUS_ on (110) facet and two‐coordinated O_CUS_ on (100) facet. Three‐coordinated O_CUS_ is less electrophilic than two‐coordinated O_CUS_, which is to the disadvantage of the breakage of the C=C bond in propylene.

**Figure 19:**
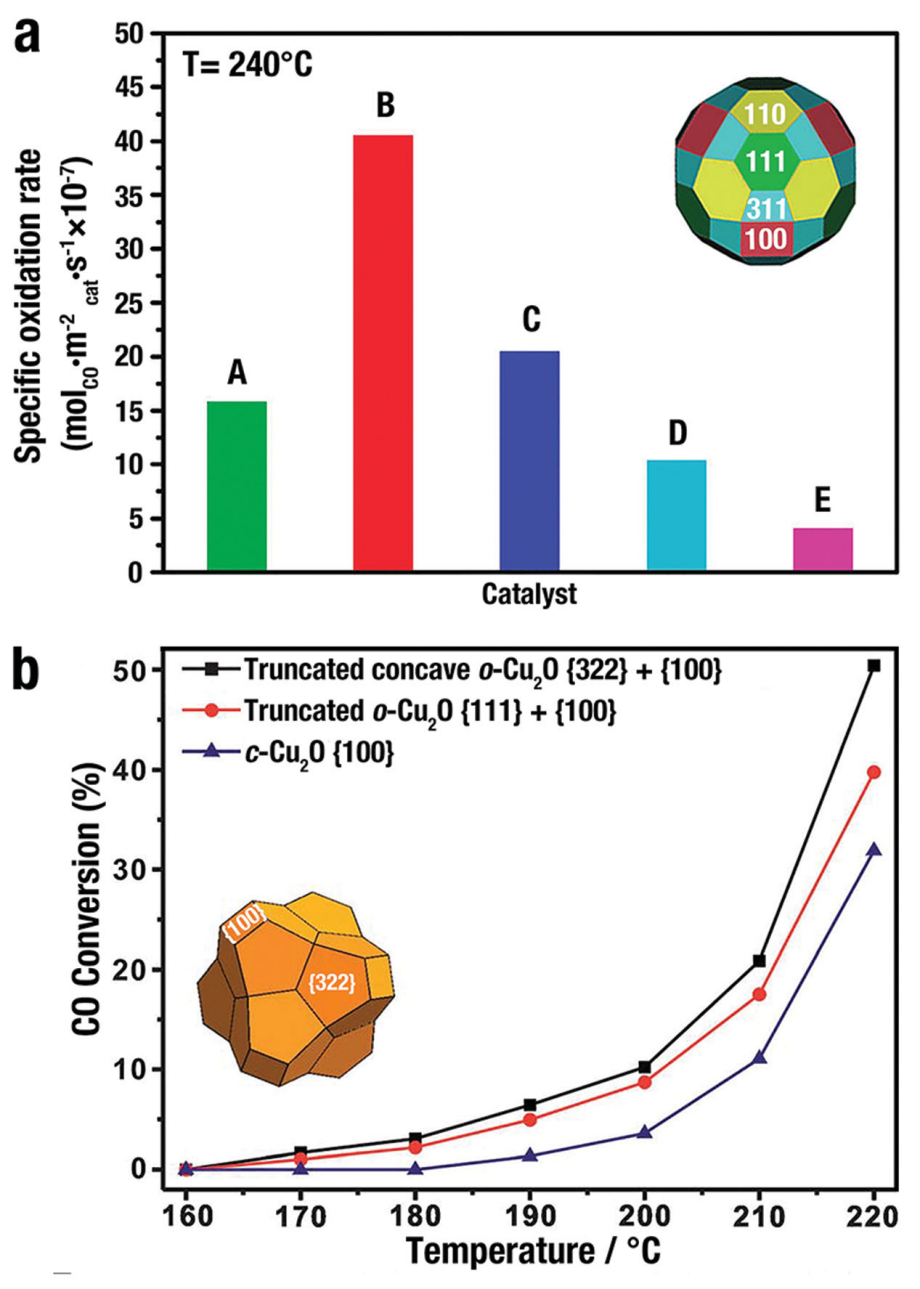
a) The specific oxidation rates of CO catalysed by different Cu_2_O polyhedra A) rhombicuboctahedron, B) Cu_2_O 50‐facet, C) *d*‐Cu_2_O, D) *o*‐Cu_2_O, and E) *c*‐Cu_2_O at 240 °C. Reproduced with permission.^17^ Copyright 2010, American Chemical Society. b) CO conversion of Cu_2_O microcrystals of different shapes. Reproduced with permission.^121^ Copyright 2013, Royal Society of Chemistry.

Compared to low‐index facets, the high‐index facets have higher catalytic activities due to the more atomic steps and kinks. C. Wang et al.^17^ evaluated the CO oxidation of a series of Cu_2_O polyhedra. Due to the presence of high‐index {311} planes on their surfaces, the 50‐facet Cu_2_O microcrystal showed the highest specific catalytic rate (8.8 × 10^−6^ mol m^−2^_cat_ s^−1^), which was markedly higher than *d*‐Cu_2_O, *o*‐Cu_2_O, *c*‐Cu_2_O and rhombicuboctahedron (**Figure** **19**a). Z. X. Xie et al.^121^ investigated the CO oxidation of truncated concave *o*‐Cu_2_O enclosed mainly by high‐index surfaces {332} facets with high‐density atomic steps (Figure 19b, inset). Truncated concave *o*‐Cu_2_O {332} + {100} displayed the highest catalytic activity, which became active at 170 °C and reached a CO conversion rate of 50.4% at 220 °C. Truncated *o*‐Cu_2_O {111} + {100} became active at 170 °C with a lower CO conversion, and reached a CO conversion of 39.8% at 220 °C. However, *c*‐Cu_2_O illustrated the lowest catalytic activity that *c*‐Cu_2_O only became active at 190 °C and reached a CO conversion of 31.9% at 220 °C (Figure 19b).

### 6.3 Organocatalysis

Numerous important products (optical devices, drugs, materials, etc.) commercialized or in the stage of development, have aromatic C—N and aromatic C—C bonds that can be coupled by organocatalysts through cross‐coupling reactions.^171^ Thus, scaling up production of these bonds with any novel and basic technology is greatly significant for industry.^171^ Over the last decade, the research focus on coupling of C—N and C—C bonds has gradually moved from the high‐cost Pd‐catalyst to the low‐cost Cu‐catalyst.^172^, ^173^ Recently, Cu_2_O (NC form or bulk) has been reported as excellent catalysts for cross‐coupling reactions.^14^, ^24^, ^32^, ^40^, ^66^ The facet‐dependent organocatalysis activity of *c*‐Cu_2_O, *o*‐Cu_2_O, and *d*‐Cu_2_O NCs was firstly evaluated by M. H. Huang et al.^24^ based on the synthesis of 1,2,3‐triazoles^14^ and the regioselective synthesis of 3,5‐disubstituted isoxazoles.^24^ To compare the catalytic activities of each Cu_2_O NCs, all the three NCs were used with identical surface area (**Table** **1**). *d*‐Cu_2_O displayed the most efficient catalytic activity, with shortest reaction times and the highest product yields, followed by *o*‐Cu_2_O and the least active *c*‐Cu_2_O. These results demonstrate that delicate facet controlling of Cu_2_O NCs can greatly improve the organocatalytic efficiency. Subsequently, M. H. Huang et al.^32^ developed a capping‐free synthetic approach for the synthesis of sub‐100 nm Cu_2_O NCs with morphology evolution from *c*‐Cu_2_O to *o*‐Cu_2_O. All the Cu_2_O NCs illustrated high yields within short reaction times. *o*‐Cu_2_O was the most excellent catalyst that could catalyse the cycloaddition reaction in just 2 h with high yields.

**Table 1:**
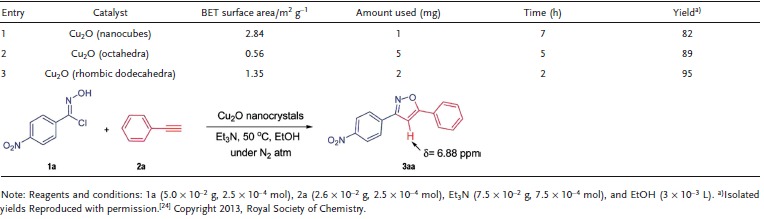
The catalytic abilities of Cu_2_O NCs for the synthesis of 3‐(4‐nitrophenyl)‐5‐phenylisoxazolea

**Figure 20:**
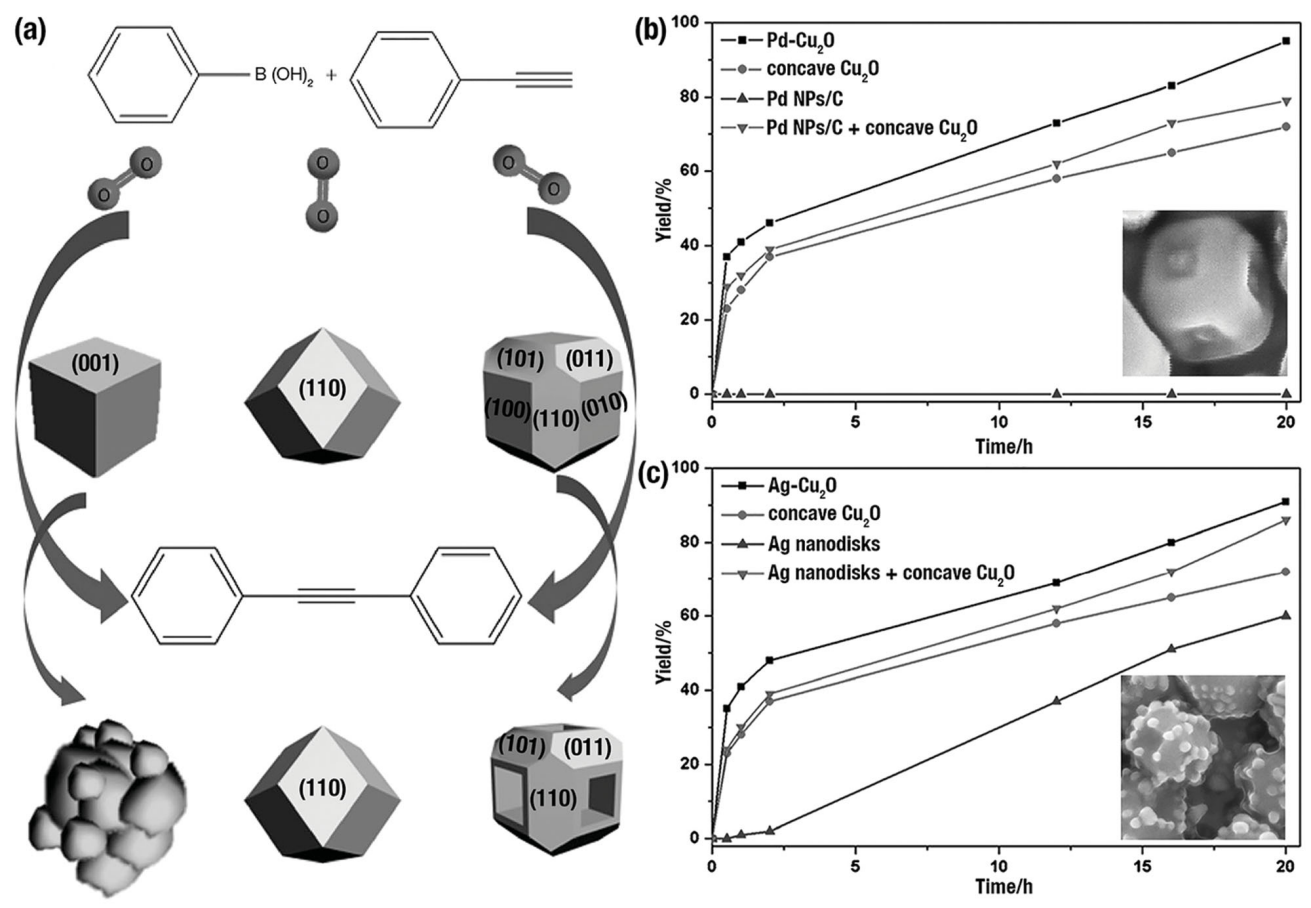
a) Shape evolution of the three different Cu_2_O NCs during the aerobic oxidative arylation of phenylacetylene. Reproduced with permission.^66^ Catalytic activities of b) Pd‐Cu_2_O and c) Ag‐Cu_2_O nanoconcave in the aerobic oxidative arylation of phenylacetylene. Inset of (b) and (c) is the typical SEM images of Pd‐Cu_2_O and Ag‐Cu_2_O, respectively. Reproduced with permission.^40^

L. L. Li et al.^66^ employed monodisperse *c*‐Cu_2_O, *d*‐Cu_2_O, and octadecahedra to catalyse aerobic oxidative coupling of phenylacetylene and arylboronic acids. During the catalytic reaction, those NCs showed high yields but different crystal surface stability. After three catalytic cycles, *c*‐Cu_2_O were seriously etched and aggregated beyond recognition, as well as their yield dropping from 94% to 32%. By contrast, no change was observed in the {110} facets of *d*‐Cu_2_O and octadecahedra during the reaction. Interestingly, Cu_2_O octadecahedra had the best catalytic activity upon recycling, and the yield of aerobic oxidative coupling was increased from 89% to 97%. The reason was that the {100} facets of Cu_2_O octadecahedra were more prone to etching than the {110} facets during this catalytic reaction, and the Cu_2_O octadecahedra were gradually oxidized and etched to high‐active concaves (**Figure** **20**a). However, the in‐depth etching mechanism still requires further study. A metal–metal oxide interface formed after deposition of noble metal onto metal oxide, and the hybrid structure displayed superior catalytic performances to the physical mixtures or single domains.^30^, ^33^, ^36^, ^40^, ^50^, ^145^ L. L. Li et al.^40^ further improved the experimental route by selective depositing noble metals on the concave Cu_2_O NCs. Pd atoms only grew on cavities (Figure 20b, inset), but Ag^0^ majorly nucleated on edges and vertices (Figure 20c, inset). During the aerobic oxidative arylation of phenylacetylene, the hybrid nanoconcaves exhibited more excellent catalytic activities than the single component or physical mixtures (Figure 20b,c). XPS spectra combined DFT calculation results verified the improvement of catalytic activities attributed to the synergistic effect, in which e^−^ migrated from the noble metal to Cu_2_O.

### 6.4 Sensing

**Figure 21:**
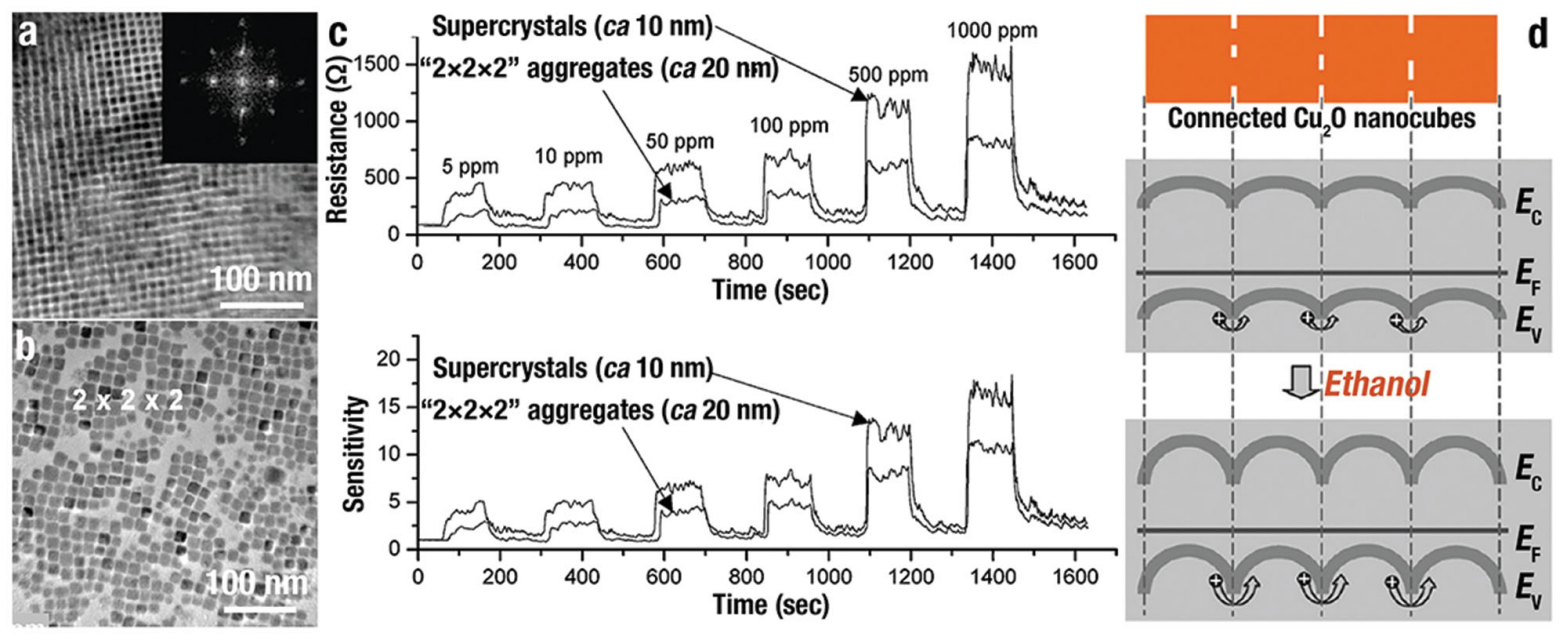
TEM images of a) Cu_2_O self‐assembled 3D supercrystals and b) disassembled nanocubes. c) Their corresponding sensitivities toward ethanol sensing measured under identical situations. d) The schematic diagrams of 1D array of *c*‐Cu_2_O toward ethanol sensing, where *E*_F_, *E*_V_, and *E*_C_ are Fermi energy, valence band energy, and conduction band energy, respectively. Reproduced with permission.^8^ Copyright 2010, American Chemical Society.

H. C. Zeng et al.^8^ evaluated the ethanol sensing ability of Cu_2_O self‐assembled 3D superlattices (≈10 nm) and disassembled nanocubes (≈20 nm), and the corresponding TEM images were shown in **Figure** **21**a, b, respectively. The organized Cu_2_O illustrated a better sensing capability than the disassembled Cu_2_O (Figure 21c). Without ethanol mole­cules, the defects on the surface of Cu_2_O would adsorb O_2_ from air and negatively charged O^−^, O_2_^−^, and O^2−^ are produced. A localized accumulation of holes were formed that separated from e^−^ near the surfaces of Cu_2_O. When Cu_2_O was exposed to ethanol/air mixtures, e^−^ was produced from the redox reactions between adsorbed O_2_ on the surface and ethanol that would be transferred into conduction band of Cu_2_O. And then, e^−^ and h^+^ would be recombined that lead to decrease the concentration of carrier. Due to the higher ratio of surface to bulk, a greater carrier depletion layer would be formed of the self‐assembled *c*‐Cu_2_O when exposed to ethanol. That layer, in cooperation with a relatively small contact potential, would result in a distinct improvement of sensitivity (Figure 21d).

**Figure 22:**
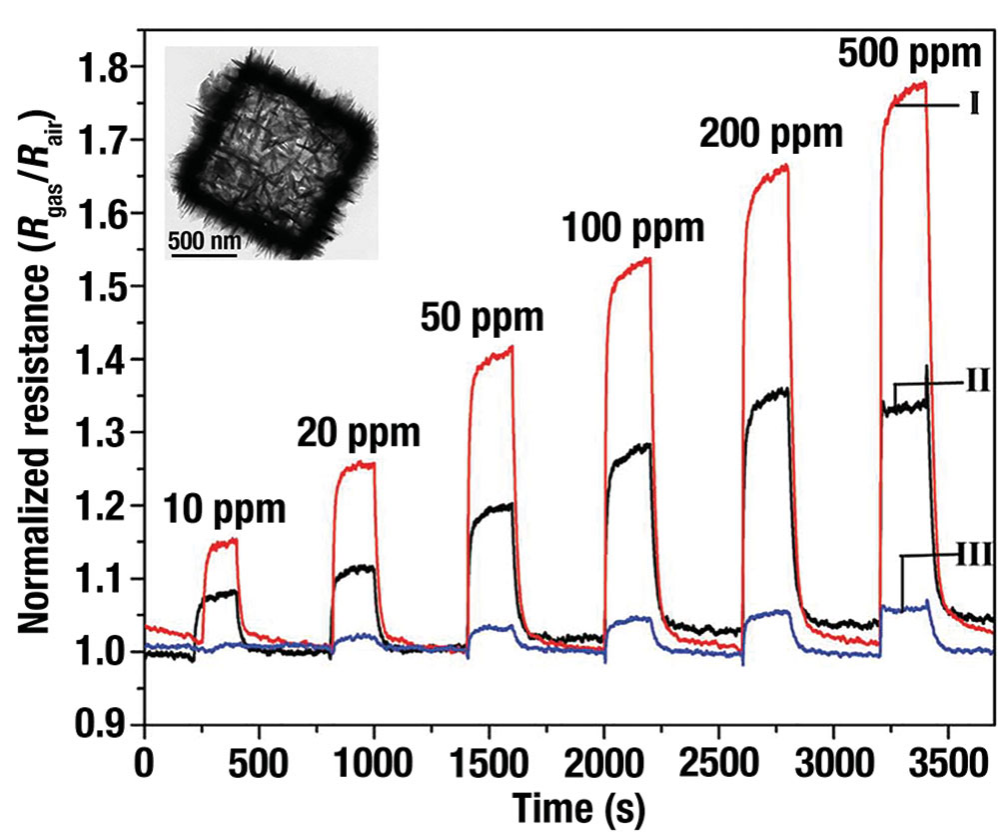
The normalized resistance of CO exposure on (I) Cu_2_O‐CuO microframes, (II) pure CuO cubes and (III) pure Cu_2_O cubes. Inset is a typical TEM image of the Cu_2_O‐CuO microframe. Reproduced with permission.^60^ Copyright 2013, Royal Society of Chemistry.

Our group^60^ evaluated the CO sensing performance of Cu_2_O–CuO composite microframes at the working temperature of 240 ºC. As the concentration of CO increased, the Cu_2_O–CuO composite microframes illustrated excellent CO sensing performance with highest sensitivity and shortest response time, followed by the pure CuO microcubes and the pure Cu_2_O microcubes (**Figure** **22**).

**Figure 23:**
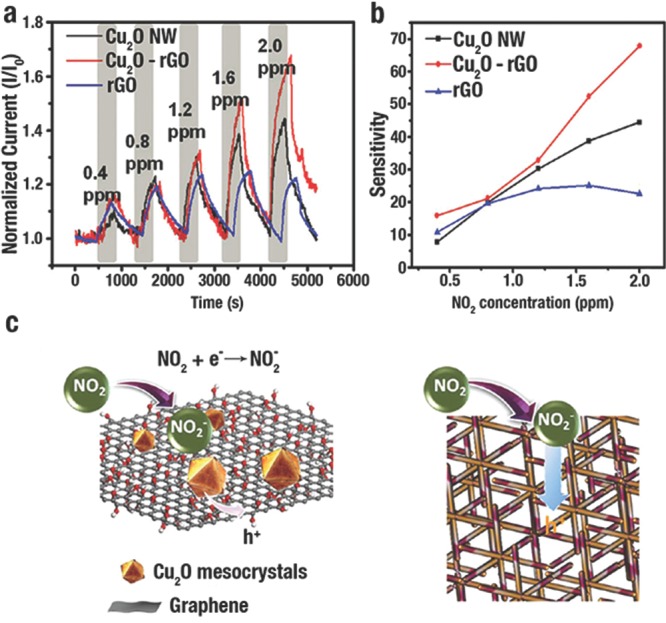
a) Response of Cu_2_O NW, rGO‐Cu_2_O, and rGO with the increasing concentration of NO_2_. b) The sensitivities for detection of NO_2_ on Cu_2_O NW, rGO‐Cu_2_O, and rGO. c) The mechanism of rGO‐Cu_2_O toward NO_2_ sensing. Reproduced with permission.^81^ Copyright 2012, American Chemical Society.

In the presence of o‐anisidine and graphene oxide, H. M. Fan et al.^81^ synthesized reduced graphene oxide (rGO)–conjugated Cu_2_O nanowire (NW) composite mesocrystals. The obtained mesocrystals with marked octahedral shape and eight {111} facets were composed of highly oriented nanowires. They further compared the NO_2_ sensing performance of Cu_2_O NW, rGO and rGO–Cu_2_O mesocrystals at room temperature. In the presence of NO_2_, all the three samples exhibited an increased sensitivity with the increasing concentration of NO_2_ (**Figure** **23**a,b). The sensitivities of rGO–Cu_2_O, Cu_2_O NW, and rGO at 2.0 ppm were 67.8%, 44.5%, and 22.5%, respectively. And the limits of detection (LOD) were calculated as 64, 81, and 82 ppb for rGO–Cu_2_O, Cu_2_O NW, and rGO, respectively. The improved sensing performance of the rGO–Cu_2_O mesocrystals was attributed to their high specific surface area and enhanced conductivity. When rGO–Cu_2_O was exposed to NO_2_, the NO_2_ molecule could obtain e^−^ from the “activated” surface O ion, and the rGO with excellent electrical conductivity could effectively transfer electrons that promoted the h^+^ conductivity in the Cu_2_O (Figure 23c). Furthermore, because of the porous and highly anisotropic structure of NW mesocrystals cooperated with the rGO, the rGO–Cu_2_O possessed larger surface accessibility for contacting NO_2_.

**Figure 24:**
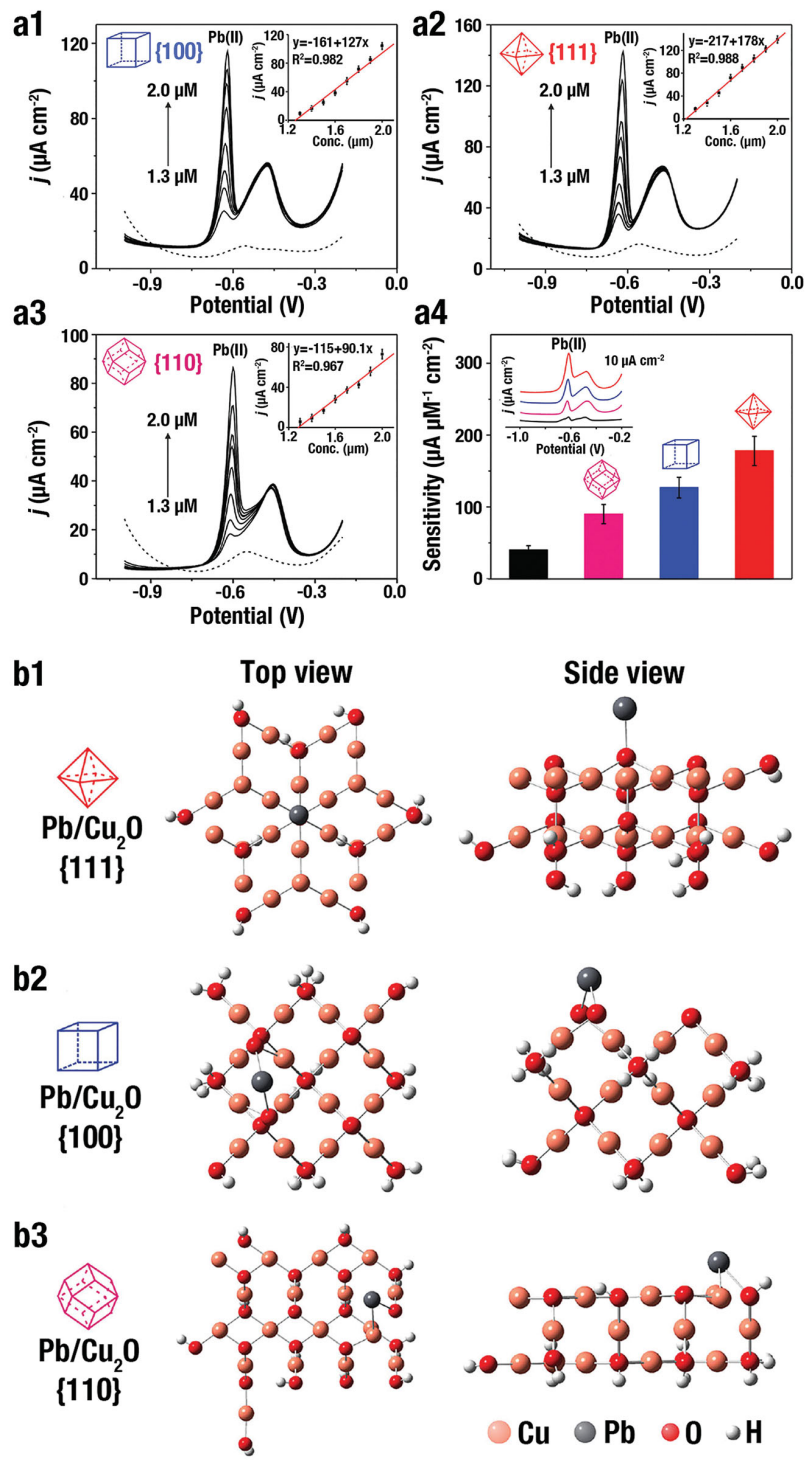
a1–a4) Typical SWASV responses of a1) *c*‐Cu2O, a2) *o*‐Cu2O, and a3) *d*‐Cu2O to detect Pb(II) in optimal situations. The corresponding linear fitting was inset in (a1), (a2) and (a3), respectively. a4) Sensitivities for SWASV detection of Pb(II) on bare GCE, *d*‐Cu2O, *c*‐Cu2O, and *o*‐Cu2O modified GCE. Inset of (a4) shows SWASV responses of 1.4 × 10^−6^ m Pb(II) on bare GCE (black line), *o*‐Cu2O (red line), *c*‐Cu2O (blue line), and *d*‐Cu2O (pink line) modified GCE. b1–b3) Top and side views of optimized adsorption models of Pb(II) on different Cu2O NCs simulated by DFT. Reproduced with permission.^29^

Recently, S. H. Yu et al.^29^ investigated the facet‐dependent stripping behaviour in the determination of Pb^2+^ by using *c*‐Cu_2_O, *o*‐Cu_2_O, and *d*‐Cu_2_O through square wave stripping voltammetry (SWASV). At –0.65 V, all three samples illustrated sharp stripping peaks of Pb^2+^ with different detection limit and sensitivity (**Figure** **24**a). The *o*‐Cu_2_O modified electrode illustrated the lowest detection limit of 0.066 × 10^−6^ M (Figure 24a2) and highest sensitivity of 178 ± 20.3 μA μm^−1^ cm^−2^ (Figure 24a4), followed by *c*‐Cu_2_O of 0.076 × 10^−6^ M (Figure 24a1) and 127 ± 14.4 μA μm^−1^ cm^−2^ (Figure 24a4), and *d*‐Cu_2_O of 0.103 × 10^−6^ M (Figure 24a3) and 90.1 ± 13.4 μA μm^−1^ cm^−2^ (Figure 24a4). It suggested that the order of stripping response of Pb^2+^ on Cu_2_O microcrystal facets was found to follow the sequence {111} > {100} > {110}.

DFT calculations were employed to investigate the adsorption mechanism of Pb^2+^ on Cu_2_O. Figure 24b depicted the conditions of Pb^2+^ adsorption on different Cu_2_O surfaces. When Pb^2+^ adsorbed on {111} surface (Pb/Cu_2_O {111}) (Figure 24b1), one O atom coordinated with Pb^2+^, and the distance of Pb–O bond was 2.188 Å. When for the {100} surface (Figure 24b2), two O atoms coordinated with Pb^2+^, and the distance of Pb–O bond was 2.260 and 2.223 Å. Figure 24b3 showed the Pb/Cu_2_O {110} that one Cu atom and one O atom coordinated with Pb^2+^, because the Cu and O atoms were in the identical plane in top layer, and the distances of Pb–Cu and Pb–O bond were 2.685 Å and 2.183 Å. The shorter distance of Pb–O in Pb/Cu_2_O {111} contributed to the strong adsorption with the Cu_2_O surface. Furthermore, the adsorption Gibbs free energy of Pb(II) on {100}, {110} and {111} facets were calculated as 4.952, 4.761 and 5.742 eV, respectively. These calculated results demonstrate the stronger Pb^2+^ adsorption ability of {111} facet, followed by the {100} and {110} facet, which agreed quite well with the electrochemical performance.

## 7 Conclusion and outlook

Through numerous examples, we have demonstrated that facet‐controlled synthetic strategies provide remarkably facile and convenient approaches to the preparation of Cu_2_O‐based NCs with heterogeneous, etched, or hollow structures. These routes depend on the different surface atomic structure of Cu_2_O NCs, in which the selective adsorption of CAs could protect special facets, and the surface energy and active sites would determine the reaction activity trend. The facet‐dependent properties of the Cu_2_O NCs and such Cu_2_O‐based NCs have been investigated, especially in the realm of photocatalysis, gas catalysis, organocatalysis and sensing. Due to different crystal surface structures, the Cu_2_O NCs exhibit distinct facet‐dependent properties; a subsequently rational design and synthesis of Cu_2_O‐based NCs could tailor and optimize their facet‐dependent performances. Although the controllable synthesis of NCs and their derivatives has seen considerable progress and development in the last decade, the next requirements for NCs with excellent performance are the development of more simple and convenient synthetic routes to tailor NCs with ideal components and structures.

The progress we have summarized also opens the door for in‐detail studies in catalysis and sensing. Due to their well‐defined facets, shape‐controlled NCs can provide smooth surfaces for further delicate carving, modifying, or transforming; a deep insight into the relationship between structures and properties will be obtained by combining with theoretical calculations and simulations of the catalysis and sensing process. This perception will enable further delicate tailoring of NCs, and bridge the gap between structures and properties, so that traditional trial‐and‐error pattern to obtain functional NCs would be instead replaced by ingenious design and controllable synthesis.

Finally, it is should be envisaged that the facet‐dependent properties of Cu_2_O‐based NCs could also apply to other realms. For instance, our group^92^ found that *o*‐Cu_2_O NCs displayed a higher oxidative stress to *D. magna* than that of *c*‐Cu_2_O due to its higher reactivity. M. H. Huang et al.^9^ investigated the facet‐dependent electrical properties of the three basic Cu_2_O NCs. *o*‐Cu_2_O is highly conductive, *c*‐Cu_2_O is moderately conductive, and *d*‐Cu_2_O is nonconductive. A thin surface layer having different degrees of band bending contributed to the different conductivities. Interestingly, a diode‐like response was obtained when electrical connection was made on two different facets of a rhombicuboctahedron. D. F. Xue et al.^25^ evaluated Li‐ion battery anode performances and showed that *c*‐Cu_2_O had the highest capacity among Cu_2_O polyhedra (the sequence of electroactivity is {110} < {111} < {100}), because {100} facets had high electroactivities toward redox reactions. Thus, more instances of the facet‐dependent properties should be continuously explored, endowing nanomaterials with excellent performances for numerous applications.
